# Dopamine Levels Induced by Substance Abuse Alter Efficacy of Maraviroc and Expression of CCR5 Conformations on Myeloid Cells: Implications for NeuroHIV

**DOI:** 10.3389/fimmu.2021.663061

**Published:** 2021-05-19

**Authors:** Stephanie M. Matt, Emily A. Nickoloff-Bybel, Yi Rong, Kaitlyn Runner, Hannah Johnson, Margaret H. O’Connor, Elias K. Haddad, Peter J. Gaskill

**Affiliations:** ^1^Department of Pharmacology and Physiology, Drexel University College of Medicine, Philadelphia, PA, United States; ^2^Division of Infectious Diseases and HIV Medicine, Drexel University College of Medicine, Philadelphia, PA, United States; ^3^Department of Medicine, Drexel University College of Medicine, Philadelphia, PA, United States

**Keywords:** neuroHIV, macrophage, microglia, dopamine, drug abuse, CCR5, maraviroc

## Abstract

Despite widespread use of antiretroviral therapy (ART), HIV remains a major public health issue. Even with effective ART many infected individuals still suffer from the constellation of neurological symptoms now known as neuroHIV. These symptoms can be exacerbated by substance abuse, a common comorbidity among HIV-infected individuals. The mechanism(s) by which different types of drugs impact neuroHIV remains unclear, but all drugs of abuse increase central nervous system (CNS) dopamine and elevated dopamine increases HIV infection and inflammation in human myeloid cells including macrophages and microglia, the primary targets for HIV in the brain. Thus, drug-induced increases in CNS dopamine may be a common mechanism by which distinct addictive substances alter neuroHIV. Myeloid cells are generally infected by HIV strains that use the chemokine receptor CCR5 as a co-receptor, and our data indicate that in a subset of individuals, drug-induced levels of dopamine could interfere with the effectiveness of the CCR5 inhibitor Maraviroc. CCR5 can adopt distinct conformations that differentially regulate the efficiency of HIV entry and subsequent replication and using qPCR, flow cytometry, Western blotting and high content fluorescent imaging, we show that dopamine alters the expression of specific CCR5 conformations of CCR5 on the surface of human macrophages. These changes are not affected by association with lipid rafts, but do correlate with dopamine receptor gene expression levels, specifically higher levels of D1-like dopamine receptors. These data also demonstrate that dopamine increases HIV replication and alters CCR5 conformations in human microglia similarly to macrophages. These data support the importance of dopamine in the development of neuroHIV and indicate that dopamine signaling pathways should be examined as a target in antiretroviral therapies specifically tailored to HIV-infected drug abusers. Further, these studies show the potential immunomodulatory role of dopamine, suggesting changes in this neurotransmitter may also affect the progression of other diseases.

## Introduction

While antiretroviral therapy (ART) has been broadly successful, HIV infection remains a global health crisis and HIV-infected individuals are still vulnerable to a wide array of comorbid diseases. Among these are a collection of neurological sequelae, collectively known as neuroHIV, which remain prevalent in infected individuals (1–4). NeuroHIV can be altered and exacerbated by substance abuse (5–8), one of the most common comorbidities in the HIV-infected population (8–15). Substance abuse is associated with altered neuropathology, increased neuroinflammation, cognitive decline and increased neuropsychiatric comorbidities, even with effective ART (16–26). However, the mechanism(s) by which distinct substances of abuse exacerbate these symptoms are unclear. Therefore, delineating these mechanisms is critical to the development of therapies that ameliorate the impact of substance abuse on neuroHIV and other comorbid neuropathologies (27, 28).

Abused substances can dysregulate immune function and increase HIV replication in myeloid cells such as macrophages and microglia (17, 18, 29–31), the primary central nervous system (CNS) targets for HIV infection (32–35). Abused substances can influence CNS myeloid cells by acting directly through surface receptors such as TLR4 (36, 37), or by altering the release of neurotransmitters, immunomodulatory and cytotoxic factors to which myeloid cells could be exposed (38, 39). These effects are prominent in dopaminergic brain regions (40, 41), and in HIV-infected individuals, neuropathology, neuroinflammation and levels of viral replication are elevated in dopamine-rich regions relative to non-dopaminergic areas (35, 42, 43). Different classes of drugs have distinct mechanisms of action, but the use of all addictive substances induces the production of the neurotransmitter dopamine in the mesocorticolimbic system (44, 45). Commonly studied as a central component of the reward or motor pathways, increasing evidence indicates that dopamine also regulates immune function (46–51). Immune cells in the CNS and periphery express all five subtypes of dopamine receptors (DRD1, 2, 3, 4, 5) and other dopamine-related proteins, enabling dopamine to regulate a variety of immune functions in both homeostatic and pathological conditions (52, 53). Exposure to dopamine concentrations induced by substance abuse increases HIV replication by increasing the number of infected macrophages (54–56). Further, dopamine has been shown to alter a variety of other functions that differ by cell type but include modulation of cytokine and chemokine secretion, changes in phagocytic activity, proliferation and chemotaxis (50, 57–60).

Drug abuse is also associated with delayed viral suppression after ART initiation and increased frequency of drug resistance mutations in HIV-infected individuals (61–64). HIV-infected individuals with methamphetamine in their system show increased plasma virus loads only if they were receiving ART, suggesting that recent drug use and ART can interact (65). The mechanistic connection between substance abuse and the HIV progression is not clear, but one connection could be through changes in the HIV co-receptor CCR5. This chemokine receptor generally mediates the entry of HIV virions into myeloid cells such as macrophages or microglia (66, 67), and our prior data show that the impact of dopamine on HIV infection requires CCR5 (55). In addition, both methamphetamine and cocaine increase CCR5 expression in non-human primate (68, 69) and rodent models of substance abuse (70). Cocaine also produces place preference and locomotor activation that are reduced by the ART drug maraviroc (MVC), a CCR5 inhibitor (70). The promoter region of CCR5 has binding sites specific to dopamine-responsive transcription factors (71) and CCR5 deficiency in mice induces both a loss of dopaminergic neurons and microglial activation (72). These and other data indicate that CCR5 expression and function could be altered in HIV-infected substance abusers and suggest a bidirectional interaction between dopamine and CCR5 in the formation of drug-associated behaviors.

The CCR5 receptor exists in several durable, antigenically distinct subpopulations within the plasma membrane (73–75), each representing different physical conformations of CCR5. Changes in conformation regulate the accessibility or binding affinity of certain CCR5 regions to different ligands, altering processes such as receptor endocytosis, G-protein signaling and HIV entry (73, 74). Most of these conformational changes alter binding affinities for either the 2^nd^ extracellular loop (ECL2) or N-terminal (NT) regions of the receptor. These domains are central to receptor interactions with both endogenous ligands (76) and the HIV envelope protein gp120 (77). The distinct conformational subpopulations of CCR5 differentially colocalize to lipid raft regions of the plasma membrane (75), which are important to receptor function (78). Critically, even small changes in CCR5 surface expression mediate distinct biological effects (79–82), so factors that alter the relative proportions of distinct CCR5 conformations could have an outsized biological impact. However, the stimuli mediating conformational shifts in CCR5 are not well understood. Our previous studies indicate that dopamine does not change the surface expression of the CCR5 conformation exposing the ECL2 region (ECL2 CCR5) (55), but in human THP-1 myeloid cells dopamine increases the surface expression of CCR5 exposing the NT region (NT CCR5) (83). This suggests drug-induced increases in dopamine could alter the expression, conformation and/or localization of CCR5 on myeloid cells, altering both the spread of HIV infection and therapeutics that specifically target the viral entry process.

To address this, we examined the impact of drug-induced dopamine levels on HIV infection and CCR5 expression and conformation in both human macrophages and microglia. Our data show that dopamine has bimodal effects on the CCR5 inhibitor Maraviroc on HIV infection in human monocyte-derived macrophages (hMDM), reducing its effectiveness in hMDM from some individuals and enhancing its effectiveness in others. Genetic analyses show that dopamine receptor expression significantly correlates with CCR5 expression in hMDM. Analysis of specific CCR5 conformations on the hMDM surface demonstrate that short term dopamine significantly increases the expression of the CCR5 conformation exposing the NT CCR5 region, and that more long term exposure to dopamine increases both NT and ECL2 CCR5. High-content imaging across hMDM populations indicates that dopamine can increase the number of individual cells expressing higher amounts of NT and ECL2 CCR5. Additionally, dopamine-mediated increases in both HIV infection and NT CCR5 expression were seen in iPSC-derived microglia and a human microglial cell line. These data demonstrate that dopamine levels induced by substance abuse increase HIV infection and can alter effectiveness of ART targeting CCR5, potentially through changes in the surface expression of different CCR5 conformations in multiple types of myeloid cells.

## Methods

### Reagents

RPMI-1640 and DMEM media, sodium pyruvate, trypsin, penicillin/streptomycin (P/S) and TrypLE were from Invitrogen (ThermoFisher, Carlsbad, CA, USA). Bovine serum albumin (BSA) and glycine were from Fisher Scientific (Waltham, MA, USA). Tween, dimethyl sulfoxide (DMSO), and hydroxyethyl piperazineethanesulfonic acid (HEPES) were obtained from Sigma-Aldrich (St. Louis, MO, USA). Fetal calf serum (FBS) was from Corning (cat # MT35010CV) and human AB serum was from Gemini Bio-Products (cat # 100-512). Paraformaldehyde (16%) was from Electron Microscopy Sciences (cat # 50980488). Macrophage colony stimulating factor (M- CSF), IL-34, TGF-β1, and IL-10 were from Peprotech (Rocky Hill, NJ, USA). The CCR5 inhibitor, Maraviroc (cat #11580) was obtained through the NIH AIDS Reagent Program, Division of AIDS, NIAID, NIH. Maraviroc was diluted to a stock concentration of 10 mM in DMSO and stored at -80°C prior to use. Dopamine hydrochloride (DA), from Sigma-Aldrich, was resuspended in diH_2_O as a 10 mM stock and stored at -20°C prior to use. All dopamine treatments were performed in the dark using 10^-6^M dopamine, unless otherwise noted, as this is the concentration of dopamine to which CNS myeloid populations could be exposed to during the abuse of substances such as cocaine and methamphetamine (52). Dopamine can oxidize and form reactive oxygen species *in vitro* (84, 85), but our previous data show that the impact of dopamine on HIV infection of macrophages is not affected by dopamine oxidation (54).

### Generation of Primary Macrophages From Human Donors

Human peripheral blood mononuclear cells (PBMC) were separated from blood obtained from de-identified healthy donors (New York Blood Center, Long Island City, NY, USA or the University of Pennsylvania Human Immunology Core, Philadelphia, PA, USA) by Ficoll-Paque (GE Healthcare, Piscataway, NJ, USA) gradient centrifugation. PBMC were isolated and matured into monocyte-derived macrophages (hMDM) using adherence isolation. Cells were cultured for 6-7 days in macrophage media (RPMI-1640 with 10% FBS, 5% human AB serum, 10 mM HEPES, 1% P/S, and 10 ng/mL M-CSF). Limited, de-identified demographic information obtained from the New York Blood Center and Penn for each donor, including age, gender, ethnicity, blood type and CMV status are found in **Table 1**. All data categories were not available for each donor, and medication, history of surgery, alcohol use and drug use status were not available. The entire data set of 88 donors was used to determine the relative expression of dopamine receptors, but not all demographic information was disclosed for every donor so not every donor was used for every correlation. Dopamine receptor expression from subsets of these donors have been previously published (50, 56) and this study examines all donors combined from previous studies as well as new donors used in this study. Our previous studies using hMDM indicated that a large data set was needed to examine correlations with dopamine receptors due to the variability inherent in primary human macrophages (50).

**Table 1:** Demographic characteristics of donors (N=88).

Variable	Statistic
Age (years)^a^ ^b^	39.7 (16) [16-71]
Ethnicity	
Caucasian	33%
African-American	13.6%
Hispanic/Latino	12.5%
Asian	6.8%
Multi-Race	1.1%
Not disclosed	33%
Gender (% men)^b^	51.9%
Blood Type	
O+	39.8%
A+	20.5%
O-	10.2%
B+	10.2%
A-	1.1%
B-	1.1%
Not disclosed	17%
CMV status (% +)^c^	50.7 %

a Mean (standard deviation) [range].

b Statistic based on 79/88 donors.

c Statistic based on 73/88 donors.

### Differentiation and Culture of Human iPSC-Derived Microglia

The inducible pluripotent stem-derived microglia (iMicroglia) were generated from common myeloid progenitors obtained from the Human Pluripotent Stem Cell Core at the Children’s Hospital of Philadelphia (CHOP). This process used a defined 11-day differentiation protocol that produces ramified cells that are susceptible to HIV infection and express the microglial markers CX_3_CR_1_, IBA1, TMEM119, and P2RY12, with very similar gene expression to human microglia (86). The cells used in this study were derived from the WT6 iPSC cell line. These cells were differentiated and maintained in 24 or 96 well Cellbind plates (Fisher Scientific) in RPMI-1640 supplemented with 1% FBS, 0.1% P/S, and the cytokines IL-34 (100 ng/mL), M-CSF (25 ng/mL), and TGF-β1 (50 ng/mL) at 37°C in a humidified incubator under 5% CO_2_. Cytokines were added fresh with each media change. The C06 human microglial cells (87) were a generous gift from David Alvarez-Carbonell and Jonathan Karn (Case Western University). These cells were maintained in 150-cm^2^ tissue culture flasks (Falcon) in DMEM supplemented with 5% FBS, 10 mM HEPES, 1% P/S, and 1% sodium pyruvate at 37°C in a humidified incubator under 5% CO_2_.

### Viral Stocks

Viral stocks of HIV_ADA_ were generated by infecting CEM-SS cells with HIV_ADA_, a blood-derived, R5-tropic strain of HIV (88). Cell-free supernatants were collected daily from 18 to 41 days post-infection, centrifuged to remove cell debris then aliquoted and stored at -80°C for use as viral stocks. Stock concentration was determined by quantifying the amount of HIV capsid protein p24Gag (p24) per mL using an HIV p24 (high sensitivity) AlphaLISA Detection kit (Perkin-Elmer, Waltham, MA).

### Replication Assay

Human monocyte-derived macrophages (hMDM) cultured in Nunc™ MicroWell™ 96-well optical-bottom plates (Thermo Fisher Scientific, Waltham, MA) at 24,000 cells per well were inoculated in triplicate with 0.5 ng/ml HIV_ADA_ for 24 hours at 37°C. Inoculations were performed concurrently with treatment with either vehicle (DMSO), maraviroc, and/or dopamine (10^-6^M). After 24 hours, hMDM were washed and replaced with fresh macrophage media. Supernatants were collected from each well at 3 days post-inoculation. The iPSC-derived Microglia (iMicroglia) were cultured in black walled, 96-well Cellbind plates (Fisher Scientific, 0720196) at 50,000 cells per well. iMicroglia were inoculated in triplicate with 1 ng/ml HIV_ADA_ for 24 hours, concurrent with treatment with either vehicle (diH_2_O) or dopamine (10^-6^M). After 24 hours, cells were washed and cultured for 10 days, collecting supernatant and acquiring brightfield images at 10x with a Nikon Inverted Microscope Eclipse Ts2. The C06 microglial cells were cultured in 24-well plates (Fisher Scientific, 087721) at 2,500 cells per well. These cells were inoculated with 2.5 ng/ml HIV_ADA_ in triplicate concurrent with vehicle (diH_2_O) or dopamine (10^-6^M) treatment. Media was changed 48 hours post inoculation, and a fraction of the starting media was collected from each well every 24 hours starting at 48 hours post-inoculation. Viral replication in all cultures was determined by quantifying the concentration of p24 in the supernatant by AlphaLISA (Perkin-Elmer), as supernatant p24 directly corresponds to production of HIV virions.

### Quantitative RT-PCR

Total RNA was extracted from cultured cells using Trizol (Invitrogen) or the RNeasy Mini Plus^™^ kit (Qiagen), and RNA quantity and purity were determined using a NanoDropOne spectrophotometer (Nanodrop Technologies). cDNA synthesis was performed on RNA (1 μg) using the high-capacity reverse transcriptase cDNA synthesis kit (Abcam). All dopamine receptor subtypes, CCR5, and 18s (housekeeping gene) were amplified from cDNA by quantitative PCR (qPCR) on a QuantStudio 7 using gene-specific primers. TaqMan Fast Universal Master Mix, and PCR assay probes for CCR5 (Hs99999149_s1), DRD1-5 (Hs00265245_s1, Hs00241436_m1, Hs00364455_m1, Hs00609526_m1, Hs00361234_s1), and 18s (4319413E) genes were purchased from Applied Biosystems (ThermoFisher, Waltham, MA, USA).

### Flow Cytometry

Human monocyte-derived macrophages (hMDM) cultured in 6-well plates at 950,000 cells per well were treated for 1 hour or 48 hours with vehicle (diH_2_O), dopamine (10^-6^ M) or IL-10 (50 ng/mL) as a positive control (79). Following incubation, hMDM were gently detached from culture dishes using TrypLE Express (1X) for 30 minutes at 37°C and washed with FACS buffer (PBS supplemented with 1% BSA). Cells were incubated at room temperature for 10 min in F_c_ Block, then with live/dead stain (ThermoFisher, cat # L34957) for an additional 15 min at 4°C in the dark. Following incubation, cells were washed and stained with either 2D7 anti-human CCR5-PE (20 μL, BDB555993), 3A9 anti-human CCR5-PE (20 μL, BDB556042) or the isotype-matched control IgG2a-PE (20 μL, BD Biosciences, cat # 556653). These antibodies were titrated to determine optimal concentration for hMDM and have been used to study dopamine-mediated changes in surface CCR5 in myeloid cells (55, 83) and to compare ECL2 CCR5 with NT CCR5 (89). Staining was performed for 30 min in the dark at 4°C in a volume of 100 μL. After 30 min, cells were washed with FACS buffer, fixed with 500 μL 2% paraformaldehyde, filtered using BD FACS tubes with cell strainer caps (35 μm pores) and stored at 4°C protected from light. During data acquisition, doublets were excluded using forward scatter height (FSC-H) *vs.* forward scatter area (FSC-A) gating. Forward versus side scatter (FSC *vs.* SSC) was used to identify cells of interest based on size and granularity. Live-dead staining was used to exclude cell debris. Isotype controls defined background caused by nonspecific antibody binding, and percentage of CCR5 positive cells was based off of this background removal. Flow cytometric analysis of C06 cells at 1 hour was performed identically to hMDM, except that these cells were seeded in 6-well plates at 500,000 cells per well and experiments were performed 24 hours after plating. All samples were acquired on a BD LSRFortessa (BD Biosciences, Franklin Lakes, NJ). All data was analyzed using FlowJo Version 10.

### Western Blot

Human monocyte-derived macrophages (hMDM) were cultured in 6-well plates at 950,000 cells per well and C06 cells were cultured in 6-well plates at 500,000 cells per well. All hMDM used in these experiments demonstrated IL-10-mediated increases in CCR5 surface expression by flow cytometry. Both hMDM and C06 cells were incubated with vehicle (H_2_O) or dopamine (10^-6^ M) for 1 hour, washed (1X PBS) and lysed with mammalian protein extraction reagent (M-PER, Thermo Fisher Scientific, Waltham, MA), containing 1% Halt Protease and Phosphatase Inhibitor cocktail and 1% EDTA (Thermo Fisher Scientific, Waltham, MA). Lysates were sonicated with a Q125 sonicator (Qsonica, Newtown, CT) at 25% power for 5 seconds and spun down at 13,000 RPM for 10 minutes at 4°C. Lysates were stored at 4°C for 1 – 7 days, then protein concentrations were quantified using a Bicinchoninic acid assay (BCA) using the Pierce BCA Protein Assay Kit (Thermo Fisher Scientific). Lysates were diluted to a concentration of 1 – 3 µg/µL and stored at -80°C until analyzed by Western blot.

Protein lysates were separated by gel electrophoresis on Bolt Bis-Tris Plus 10% precast gels in MOPS/SDS running buffer in a Mini gel tank (Life Technologies, Carlsbad CA). Separation was performed for 120 minutes at 150V, then protein was transferred to an Immobilon PVDF membrane (EMD Millipore, Temecula, CA) at 25V for 60 minutes. To generate an internal loading control, membrane was imaged after treatment with Revert Total Protein Stain (LI-COR Biosciences, Lincoln, NE) according to the manufacturer’s instructions. Total protein stain was then removed, membranes were blocked (5% BSA at room temperature for 1 hour) then incubated overnight at 4°C in anti-CCR5 antibody (AB1889, 1:1000 in 5% BSA, EMD Millipore). Following primary antibody incubation, blots were washed (TBS with 0.1% Tween), stained with anti-rabbit IgG HRP linked antibody (CST 7074, 1:3000 in 5% milk) and incubated at room temperature for 1 hour. After secondary incubation, blots were washed and incubated in Supersignal West Pico PLUS plus Chemiluminescent Substrate (2 mL, 30 sec, ThermoFisher, 34580). Blots were imaged using an Odyssey Fc Imaging System and analyzed using Image Studio Lite (Licor Biosciences, Lincoln, NE). Target bands were normalized to total protein stain, and then each condition was compared to the vehicle control to determine fold-change in expression.

### Immunofluorescent Analysis of CCR5

Human monocyte-derived macrophages (hMDM) were cultured in Nunc™ MicroWell™ 96-well optical-bottom plates (Thermo Fisher Scientific, Waltham, MA) at 24,000 cells per well. All cells were treated with vehicle (H_2_O), IL-10, or 10^-6^M dopamine in triplicate for 1 hour. Following treatment, cells were fixed (4% PFA at room temperature for 10 minutes, 50980488, Fisher Scientific), then incubated with wheat germ agglutinin (10 µg/mL, 10 min, W32466, Thermo Fisher). The hMDM were incubated with blocking buffer (1% BSA, 0.1% Tween 20, and 22.52 mg/mL glycine in 1XPBS) for 30 minutes at room temperature. For analysis of CCR5 surface expression, cells were incubated with primary antibodies overnight at 4°C, using either 2D7 (ECL2) CCR5 antibody (BDB555991, Fisher Scientific) or primary 3A9 (NT) CCR5 antibody (BDB556041, Fisher Scientific) made in blocking buffer. Following primary incubation, hMDM were incubated with either Alexa Fluor 488 secondary antibody (A-11001, Fisher Scientific) or Alexa Fluor 546 secondary antibody (A-11003, Fisher Scientific) made in blocking buffer for 1 hour at room temperature. All cells were then stained with DAPI (D1306, Fisher Scientific) for 10 minutes. Images were acquired on the CellInsight CX7 High Content Screening Platform (CX7), an automated 7-channel confocal microscope. Ten fields per well were imaged using a 10x objective, and images were analyzed using HCS software.

For analysis of CCR5 colocalization with lipid rafts, hMDM were treated with vehicle (H_2_O) or 10^-6^M dopamine in triplicate for 1 hour, fixed and incubated with blocking buffer as just described, and then incubated with primary 2D7 (ECL2) CCR5 antibody or primary 3A9 (NT) CCR5 antibody, primary CD71 antibody (sc-32272, Santa Cruz Biotechnology Santa Cruz, CA), and anti-Flotillin-1 antibody (BDB610820, Fisher Scientific BDB610820). All primary antibody incubations were performed in blocking buffer overnight at 4°C. Following primary incubation, hMDM were washed and incubated for 1 hour at room temperature in either Alexa Fluor 568 secondary antibody (A-11004, Fisher Scientific A-11004), Alexa Fluor 488 secondary antibody, or Alexa Fluor 647 secondary antibody (A-21235, Fisher Scientific), made in blocking buffer. Cells were then stained with DAPI and imaged on the CX7. For each well, 100 field images were taken using a 40X objective at an exposure time of 0.1 seconds, and images were analyzed using HCS software. More detailed methodology for High Content imaging and analyses is included in the **Supplemental Materials**.

### Statistical Analysis

To determine the appropriate statistical tests, all data sets were evaluated by analysis of skewness and evaluation of normality to determine the distribution of the data. Extreme data points presumed to be technical outliers were identified *via* ROUT test (Q = 0.1%) and removed from analysis. *Post-hoc* analyses were performed when appropriate. In studies analyzing gene expression, all statistical tests were performed on data normalized to 2^-δC^_T_ to preserve variance. In all experiments using a positive control, changes in the positive control were not analyzed alongside the experimental condition. Therefore, while the effects mediated by the positive control IL-10 are shown on the same graph as dopamine-mediated changes, since the effects of IL-10 were analyzed separately, they are shown by the @ sign, rather than the * used to show significance in the analyses of dopamine-mediated changes. All data analysis was performed using GraphPad Prism 9.0 (Graphpad, La Jolla, CA). p < 0.05 was considered significant.

## Results

### Dopamine Alters Effectiveness of Maraviroc in HIV-Infected hMDM

**Figure 1:**
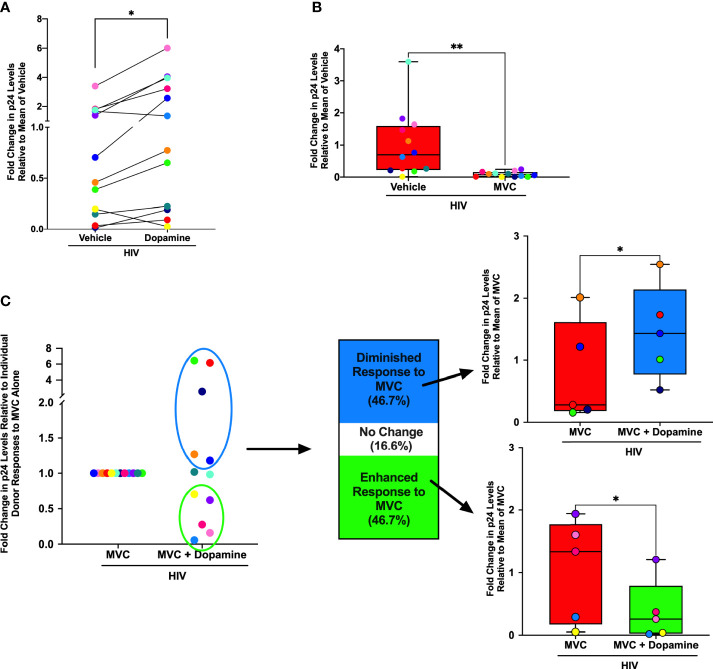
Dopamine Alters Effectiveness of Maraviroc in HIV-infected hMDM. Primary human monocyte-derived macrophages (hMDM) from twelve donors were inoculated with HIV_ADA_ (0.5 ng/mL) for 24 hours in the presence of vehicle (diH_2_O or DMSO), a dopamine (10^-6^M) condition maraviroc (MVC) (0.1 µM) or MVC + dopamine (10^-6^M). Infections were maintained in culture for 3 days, at which point supernatants were collected and examined for levels of HIV replication (p24). Responses from each donor are designated with a specific color throughout. **(A)** When examining fold change in p24 levels relative to the mean of vehicle treatment, dopamine significantly increased HIV infection alone (Paired t-test, n = 12, *p = 0.0162, t=2.835, df=11). **(B)** When examining fold change in p24 levels relative to the mean of vehicle treatment, MVC also successfully suppressed viral replication at 0.1µM, in that MVC significantly decreased p24 levels relative to HIV alone (Paired t-test, n = 12, **p = 0.008, t=3.229, df=11). **(C)** When examining fold change in p24 levels relative to individual donor responses to MVC treatment alone, there was a bimodal response to dopamine in hMDM treated with 0.1 µM MVC. Compared to the p24 levels relative to the mean of MVC alone, five out of twelve donors (46.7%) showed a dopamine-mediated diminished response to MVC (Paired t-test, n = 5, *p = 0.0391, t=3.021, df=4), and five out of twelve donors (46.7%) showed a dopamine-mediated enhanced response to MVC (Paired t-test, n = 5, *p = 0.049, t=2.796, df=4). Two of the twelve donors (16.6%) showed no response to dopamine in respect to the efficacy of MVC.

Substances of abuse can decrease the effectiveness of antiretroviral drugs (65), including maraviroc (90), the only FDA approved antiretroviral drug that targets CCR5. To determine whether dopamine was associated with this effect, hMDM from 12 donors were inoculated with HIV_ADA_ (0.5 ng/mL) for 24 hours in the presence of vehicle (diH_2_O or DMSO), dopamine (10^-6^M), maraviroc (MVC) or MVC + dopamine (10^-6^M). MVC was used at 0.1 or 1 µM, based on approximate blood molarity from c_max_ plasma values resulting from commonly prescribed doses of MVC (150 or 300 mg/day) (91, 92). Supernatant from each infection was collected on day 3 and examined for the presence of p24 as a measure of viral replication. As expected, analysis showed variations in infection between individuals (93, 94), but also showed dopamine significantly increased HIV infection alone relative to the mean of vehicle-treated, HIV-infected cells, similar to our prior data (54–56) (**Figure 1A**). Individual donors are designated with a specific color throughout **Figure 1**, showing dopamine increased p24 levels in hMDM from 10 of the 12 donors examined. We also examined whether MVC successfully suppressed viral replication, and at both 0.1 µM (**Figure 1B**) and 1µM (**Supplementary Figure 1A**), MVC significantly decreased p24 levels relative to the mean of vehicle-treated, HIV-infected cells.

To examine dopamine-mediated changes in the efficacy of MVC, we compared the mean day 3 p24 levels in HIV-infected hMDM treated with MVC to the p24 levels in HIV-infected hMDM treated with MVC and dopamine. Donors were defined as having a diminished or enhanced response to MVC if the addition of dopamine resulted in a greater than 10% change from the p24 level in the HIV+MVC condition. This analysis showed a bimodal response to dopamine in hMDM treated with 0.1 µM MVC (**Figure 1C**). hMDM from 5/12 (46.7%) donors showed a diminished response to MVC, with significantly higher levels of p24 in cultures treated with dopamine. Similarly, 5/12 (46.7%) showed an enhanced response to MVC, with relatively lower p24 levels in cultures treated with dopamine. In 2/12 donors (16.6%) dopamine did not alter the efficacy of MVC. Although not significant, similar results were obtained in the 1 µM MVC experiments (**Supplementary Figure 1B**). These data suggest that individual variations in the response to dopamine could alter the effectiveness of MVC, changing the efficacy of antiretroviral therapy in HIV-infected substance abusers.

As differences in dopamine receptor levels can contribute to donor-specific responses to dopamine (50), the donor responses to MVC and/or dopamine were compared to expression of all five subtypes of dopamine receptors (D1-like, DRD1 and DRD5; and D2-like, DRD2, 3, 4). These correlations were performed using expression of dopamine receptor transcripts by qPCR due to the lack of effective antibodies against human dopamine receptors and the inability of existing antibodies to differentiate between DRD1 and DRD5. These analyses showed no significant correlations between dopamine receptor expression and response to MVC. As age has also been shown to affect ART efficacy (95), the fold change response to dopamine and MVC was also compared to age. Although not statistically significant, the average age for the group that had a diminished response to MVC was 50.2, and the oldest donor (red dots) had the largest fold change increase in p24 levels relative to the MVC only condition. In the group that had an enhanced response to MVC, the average age was 42.8, and in this group the oldest donor had the smallest fold change decrease in p24 levels relative to the MVC only condition (yellow dots).

### Expression of CCR5 Correlates With Dopamine Receptor Expression in hMDM

**Figure 2:**
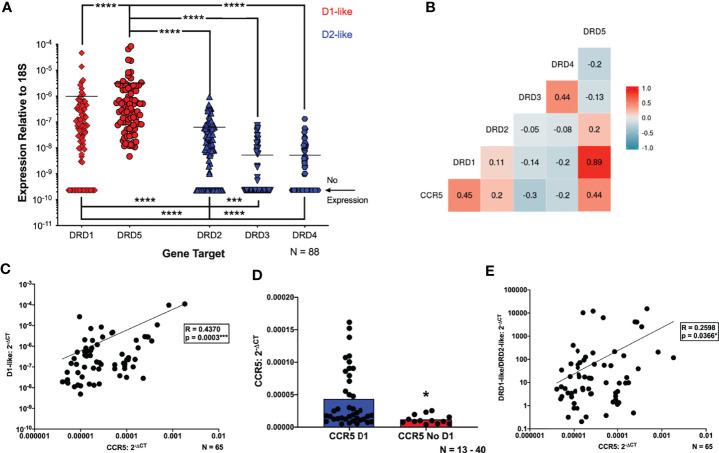
Expression of CCR5 correlates with dopamine receptor expression in hMDM. **(A)** Quantitative RT-PCR detected mRNA for all subtypes of dopamine receptors (D1R, D2R, D3R, D4R and D5R) in primary human monocyte-derived macrophages (hMDM) (N=88). Expression of all receptors was normalized to 18s for each donor. The D1-like receptors (red dots) were expressed at significantly higher levels than the D2-like receptors (blue dots) (Friedman test, n=88, Friedman statistic 233, ****p < 0.0001; *Post-hoc* with Dunn’s multiple comparisons, DRD5 *vs.* DRD1, DRD2, DRD3, or DRD4 ****p < 0.0001, DRD1 *vs.* DRD3 or DRD4, ****p < 0.0001, DRD2 *vs.* DRD3, ***p = 0.0003, and DRD2 *vs.* DRD4, ****p < 0.0001). Correlational analyses were then performed to look at correlations between each dopamine receptor and CCR5 mRNA expression (N=65). A matrix to visualize these correlations is shown in **(B)**, and we found that increased expression of CCR5 is associated with greater expression of the D1-like receptors (CCR5 vs D1, n = 65, Spearman r = 0.4454, ***p = 0.0002, CCR5 *vs* D5, n = 65, Spearman r = 0.4448, ***p = 0.0002). Increased expression of CCR5 is also associated with decreased DRD3 expression (CCR5 *vs* D3, n = 65, Spearman r = -0.3007, *p = 0.0149), and no association was found between the other D2-like receptors and CCR5. A number of donors lacked expression of one or more dopamine receptors, so the data were reanalyzed for correlations between CCR5 and the **(C)** D1-like (DRD1 and DRD5) dopamine receptors. These analyses showed a positive correlation between CCR5 and D1-like receptors (CCR5 vs D1-like dopamine receptors, n = 65, Spearman r = 0.4370, ***p = 0.0003). The connection between D1-like receptors and CCR5 was strengthened by analysis of CCR5 levels in hMDM that did or did not express DRD1. These analyses showed hMDM without DRD1 had significantly lower levels of CCR5 mRNA than those expressing DRD1 (Mann-Whitney test, n = 17 - 48, *p = 0.0218, sum of (D1, No D1) ranks 1737, 408, U=255) **(D)**. This was not done for DRD5 because all donors expressed this receptor. CCR5 levels were also correlated with a D1-like receptor/D2-like receptor ratio (D1/D2 ratio) **(E)**, and we showed a significant, positive correlation between the D1/D2 ratio and CCR5 expression (CCR5 *vs* D1/D2-like dopamine receptors, n = 65, Spearman r = 0.2598, *p = 0.0366).

Both dopamine receptors and associated proteins play a role in regulating CCR5 expression in multiple cell types (83, 96). To more precisely define the connection between the effects of dopamine and maraviroc efficacy, we examined the relationship between dopamine receptors and CCR5, using the expression levels of CCR5 and all five subtypes of dopamine receptors on uninfected hMDM from a large group of donors (N = 88) with the available demographic details shown in **Table 1**. Not all demographic details were available for every donor, so the specific numbers of donors used for each analysis are noted in the table. Gene expression analysis confirmed our previous findings showing that hMDM can express mRNA for all five subtypes of dopamine receptors, with wide variation in expression levels between donors. DRD5 is the only receptor expressed on every donor, and was significantly greater than expression of DRD1 and D2-like receptors across all donors (**Figure 2A**).

In the subset of donors for whom CCR5 expression data was available (N = 65), analyses showed a positive trend between CCR5 and age (**Supplementary Figure 2A**), corroborating other studies (97). The data also showed that females have greater CCR5 expression than males (**Supplementary Figure 2B**), which could be due to the modulation of CCR5 by sex hormones such as progesterone and estrogen (98, 99). And infection with cytomegalovirus (CMV), which is common in the adult population, can increase CCR5 expression (100), and the donors who were CMV+ had greater CCR5 expression compared to the donors that were CMV- (**Supplementary Figure 2C**).

This cohort was then used to generate a correlation matrix comparing expression between individual dopamine receptors and CCR5. There was a significant positive correlation between CCR5 and both DRD1 and DRD5 (**Figure 2B**), as well as a weaker, but still significant, negative correlation with CCR5 and DRD3 (**Figure 2B**). There were no correlations between expression of DRD2 or DRD4 and CCR5. To account for the lack of DRD3 and DRD4 expression in a number of donors, data were reanalyzed for correlations between CCR5 and either the D1-like (DRD1 and DRD5) or D2-like (DRD2, 3, 4) dopamine receptors. These analyses showed a positive correlation between CCR5 and D1-like receptors (**Figure 2C**) but no correlation between CCR5 and D2-like receptors (**Supplementary Figure 2D**).

These correlations were strengthened by examination of CCR5 expression in hMDM that did or did not express DRD1. hMDM without DRD1 showed significantly lower levels of CCR5 mRNA than those expressing DRD1 (**Figure 2D**). For D2-like dopamine receptors, there was no change in CCR5 expression in groups with or without DRD2 expression (**Supplementary Figure 2E**). Interestingly, hMDM not expressing either DRD3 or DRD4 had higher levels of CCR5 mRNA than those expressing either dopamine receptor (**Supplementary Figures 2F, G**). These analyses could not be performed for DRD5 because all donors expressed this receptor. These data suggest that both D1-like and D2-like receptors influence CCR5 expression, so CCR5 levels were correlated with a D1-like receptor/D2-like receptor ratio (D1/D2 ratio), generated by pooling the values for D1-like receptor expression and dividing them by the pooled values for D2-like expression from each donor, as has been done previously (101). Analysis showed a significant, positive correlation between the D1/D2 ratio and CCR5 expression (**Figure 2E**). Overall, these data indicate that CCR5 expression is significantly correlated with the expression of dopamine receptors, primarily D1-like receptors, on primary human macrophages.

### Dopamine Alters the Proportion of CCR5 Conformations in hMDM

**Figure 3:**
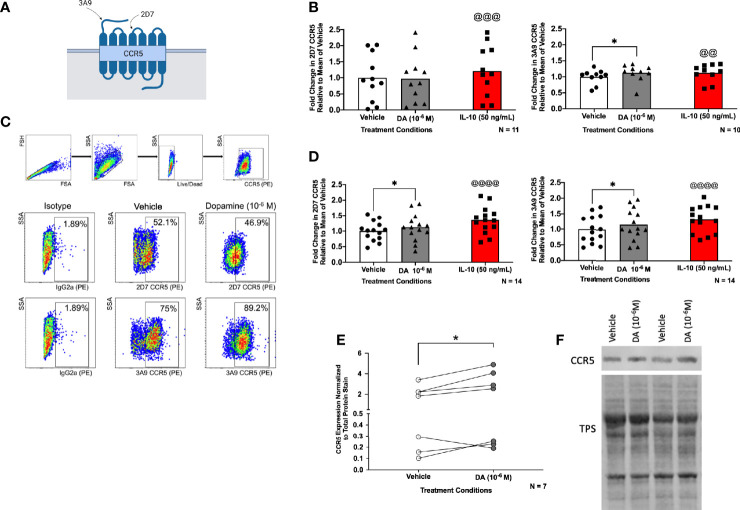
Dopamine alters the proportion of CCR5 conformations in hMDM. Surface expression of 2D7 (ECL2) and 3A9 (NT) CCR5 as depicted in **(A)** (created with BioRender.com) was analyzed by flow cytometry after hMDM were treated with vehicle (diH_2_O), dopamine (10^-6^ M), or IL-10 (50 ng/mL) as a positive control (N= 10-11, donors are not the same for each analysis) for 1 hour **(B)**. The separate statistical tests performed on the IL-10-treated samples are denoted by the use of the @ sign, rather than the * used to show significance in the analyses of dopamine-mediated changes. Fold change in CCR5 is relative to the mean of the vehicle. We found a significant increase in NT CCR5 but not ECL2 CCR5 following dopamine exposure (ECL2 CCR5, Paired t-tests, n = 11, Dopamine, p = 0.8776, t=0.1579, df=10; IL-10, ^@@@^p = 0.0009, t=4.684, df=10; NT CCR5, Wilcoxon tests, n = 10, Dopamine, *p = 0.0273, sum of (+,-) ranks 49, -6, IL-10, ^@@^p = 0.002, sum of (+,-) ranks 55, 0). **(C)** Gating strategy of hMDM by flow cytometry, and dot plot data from one representative donor, in that dopamine increases the percentage of surface NT CCR5 but not ECL2CCR5. **(D)** hMDM were also treated with vehicle (diH_2_O), dopamine (10^-6^ M), or IL-10 (50 ng/mL) as a positive control (N= 14, donors are not the same for each analysis) for 48 hours. We found a significant increase in ECL2 and NT CCR5 following dopamine exposure (ECL2 CCR5, Paired t-tests, n = 14, Dopamine, *p = 0.0296, t=2.444, df=13; IL-10, ^@@@@^p < 0.0001, t=5.936, df=13; NT CCR5, Paired t-tests, n = 14, Dopamine, *p = 0.0491 t=2.170, df=13; IL-10, ^@@@@^p < 0.0001, t=7.227, df=13). To determine whether dopamine affects N-terminal CCR5 in general, hMDM were treated with dopamine (10^-6^ M) for 1 hour and probed for CCR5. Pooled data showing fold change in hMDM from 7 donors relative to the vehicle condition is shown with CCR5 normalized to total protein stain, and dopamine significantly increased the expression of N-terminal CCR5 **(E)** (Paired t-test, n = 7, Dopamine, *p = 0.048, t=2.477, df=6). A representative blot is show in **(F)**.

To determine whether activation of dopamine receptors influences CCR5 expression, hMDM were treated with vehicle (diH_2_O), dopamine (10^-6^ M), or IL-10 (50 ng/mL) for 1 hour and 48 hours. The 1 hour time point has previously been used to assess dopamine-mediated changes in CCR5 surface expression (83), and focuses on the HIV entry process, as we have previously published that dopamine increases entry at an early timepoint (55, 56). As the maraviroc experiments showed that dopamine also influences HIV replication after 3 days in hMDM, changes in CCR5 were also examined at 48 hours. Treatment with IL-10 (50 ng/mL) was used as a positive control, as this cytokine increases CCR5 expression in human monocytes, macrophages, and microglia (79, 102, 103). This use of a positive control in hMDM is similar to what we and others have published (50, 60, 104), as there is considerable variation in the hMDM inflammatory response to environmental stimuli (105–107). Therefore, donors in which IL-10 did not increase CCR5 at 1 hour were excluded from the analysis. After 1 hour or 48 hours, cells were examined by flow cytometry for changes in expression of different surface CCR5 conformations, ECL2 CCR5 or NT CCR5 (**Figure 3A**).

Flow cytometric analysis of the pooled CCR5 expression in dopamine-treated cells after 1 hour showed a small but significant (8.71%) increase in the percentage of surface NT CCR5, almost identical to that seen in response to IL-10 (8.63%). There was no significant increase in the expression of ECL2 CCR5 after 1 hour (**Figure 3B**). A representative dot plot for the 1 hour time point is shown in **Figure 3C**. In contrast to the effect observed at 1 hour, flow cytometric analysis demonstrated that dopamine significantly increased the percentage of both surface ECL2 (12%) and NT CCR5 (16%) at 48 hours (**Figure 3D**). Notably, when there was high baseline expression, neither dopamine nor the positive control showed robust increases in CCR5 (1-5% increase), while increases in NT CCR5 were much greater when baseline NT CCR5 expression was lower (**Figure 3B**). This indicates a potential ceiling effect for this assay, suggesting the effect could be greater than reported as the potential signal saturation limited the increase in some donors. These data demonstrate that dopamine increases surface CCR5 expression and may be a part of the mechanism by which dopamine increases early viral replication and interferes with the efficacy of maraviroc.

These data were corroborated by Western blotting using a different antibody that targets the entire N-terminal region and not just a specific N-terminal epitope. In these studies, hMDM from 7 donors were treated with vehicle (diH_2_O) or dopamine (10^-6^ M) for 1 hour and then examined for CCR5. Analyzing the pooled data from all donors showed a significant, 32% increase in CCR5 expression in cells treated with dopamine relative to vehicle (**Figure 3E**). Representative blots for two donors, normalized to total protein stain (TPS) are shown (**Figure 3F**, full blots in **Supplementary Figure 3**).

**Figure 4:**
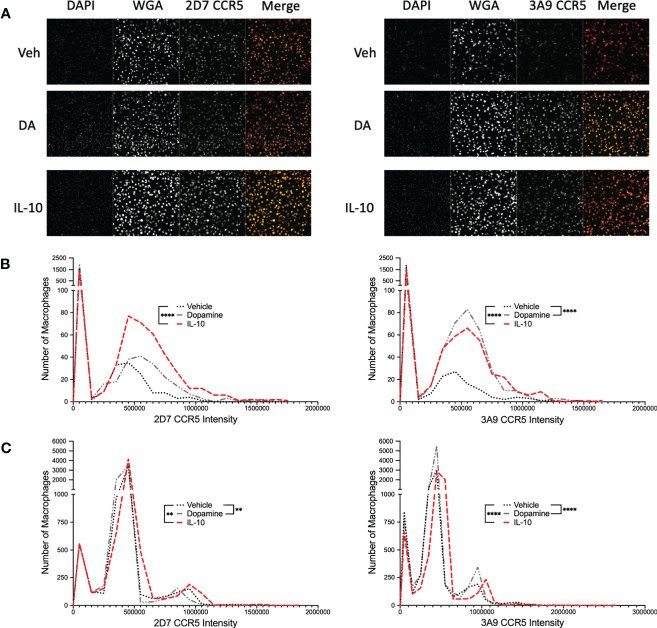
Dopamine increases the proportion of hMDM with N-Terminal CCR5. **(A)** Representative images of immunocytochemical staining of hMDM with DAPI (blue), wheat germ agglutinin (WGA) (far red) and ECL2 or NT CCR5 (red). Images were acquired on the Cell Insight CX7 automated 7-channel confocal microscope, imaging ten fields per well using a 10x objective in a 96 well plate. CX7 data from two donors were then used to generate a frequency distribution, segregating the data from each cell into bins based on intensity, with a bin size of 100,000, and graphed as a histogram, representing the number of cells contained in each bin from each experimental condition **(B)** IL-10 significantly increased the population of cells with higher levels of both 2D7 and 3A9 CCR5, while dopamine only increased the number of cells with higher levels of 3A9 CCR5 (IL-10, 2D7, ****p < 0.0001, Chi-square = 114.6, df = 1, z = 10.71, 3A9, ****p < 0.0001, Chi-square = 128.5, df = 1, z = 11.34; Dopamine, 3A9, ****p < 0.0001, Chi-square = 373.1, df = 1, z = 19.31). **(C)** IL-10 significantly increased the population of cells with higher levels of both 2D7 and 3A9 CCR5, while surprisingly dopamine also increased the number of cells with high levels of 2D7 and 3A9 CCR5 (IL-10, 2D7, **p < 0.01, Chi-square = 9.128, df = 1, z = 3.021, 3A9, ****p < 0.0001, Chi-square = 49.83, df = 1, z = 7.059; Dopamine, 2D7, **p < 0.01, Chi-square = 8.535, df = 1, z = 2.921, 3A9, ****p < 0.0001, Chi-square = 108.6, df = 1, z = 10.42).

While these data indicate that dopamine increases the percentage of hMDM expressing CCR5 across a population, they do not define whether individual hMDM also express more surface CCR5. To examine this, hMDM from two donors were treated with vehicle or dopamine for 1 hour, with IL-10 again used as a positive control. After 1 hour, hMDM were fixed and stained for cell nuclei (DAPI), cell membranes [wheat germ agglutinin (WGA)] and either ECL2 or NT CCR5, then imaged using the CX7. Representative images are shown in **Figure 4A** and data from high-content immunofluorescent imaging were used to generate a frequency distribution, segregating the data from each cell into bins based on intensity, with a bin size of 100,000. To increase accessibility, these data were graphed as a histogram, representing the number of cells contained in each bin from each experimental condition (**Figures 4B, C**). To analyze these data, we determined the 95% confidence intervals for the total population in each set of conditions (either dopamine or IL-10). Then the number of individual cells above the 95% confidence interval - representing higher levels of surface CCR5 - were enumerated for each condition and compared using a chi-squared test. For the donor in 4B, IL-10 significantly increased the population of cells with higher levels of both ECL2 and NT CCR5, while dopamine only increased the number of cells with higher levels of NT CCR5. For the donor in 4C, IL-10 significantly increased the population of cells with higher levels of both ECL2 and NT CCR5, while surprisingly dopamine also increased the number of cells with high levels of ECL2 and NT CCR5. These data demonstrate that dopamine not only altered the total number of cells with different CCR5 conformations on the cell surface, but that dopamine specifically increased the amount of CCR5 on the cells expressing a particular conformation. These findings corroborate our flow cytometry and Western blot analyses and indicate that exposure to dopamine for 1 hour could significantly change the responses mediated by CCR5 across myeloid populations.

### Dopamine Does Not Alter the Localization of Specific CCR5 Conformations Within the Plasma Membrane

Specific conformations of CCR5 preferentially localize to cholesterol-rich lipid raft microdomains within the plasma membrane (75), and in macrophages, lipid rafts are important for CCR5-mediated HIV viral entry, maintaining the conformational integrity and ligand binding activity of CCR5, and disruption of raft regions interferes with macrophage infection (108, 109). Substances of abuse can alter the translocation of receptors into lipid raft domains (110, 111) and lipid raft proteins, such as caveolin-1, can alter the function of the D1 dopamine receptor (112, 113). Therefore, high content imaging was used to assess the impact of dopamine on the localization of specific CCR5 conformations within lipid rafts in the hMDM plasma membrane.

**Figure 5:**
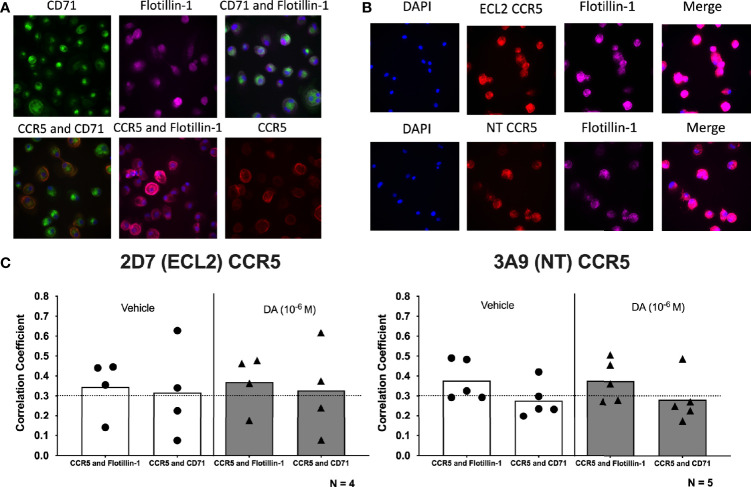
Dopamine does not alter the localization of specific CCR5 conformations within the plasma membrane. **(A)** Representative images of immunocytochemical staining of hMDM with CCR5 (red), the non-lipid raft marker CD71 (green), and the lipid raft marker flotillin-1 (purple), as well as overlay images of CCR5 and CD71 (orange), CCR5 and flotillin-1 (pink), and CD71 and flotillin-1 (blue). Images were acquired on the Cell Insight CX7 automated 7-channel confocal microscope, imaging 100 fields per well using a 40x objective in a 96-well plate. **(B)** Representative images of immunocytochemical staining of hMDM with DAPI (blue), 2D7 or 3A9 CCR5 (red), flotillin-1 (purple), and merged. **(C)** Quantitative analysis using Pearson’s correlation coefficient shows a positive correlation for 2D7 CCR5 and flotillin-1 as well as 2D7 CCR5 and CD71 in both vehicle-treated and dopamine-treated cultures ECL2 CCR5: Flotillin-1, vehicle PCC = 0.345, dopamine PCC = 0.369; ECL2 CCR5:CD71, vehicle PCC = 0.317, dopamine PCC = 0.328. In contrast, colocalization of NT CCR5 was only shown between NT CCR5 and Flotillin-1, with NT CCR5 showing no colocalization with CD71 (NT CCR5: Flotillin-1, vehicle PCC = 0.377, dopamine PCC = 0.376; NT CCR5:CD71, vehicle PCC = 0.277, dopamine PCC = 0.283).

hMDM were treated with either vehicle (diH_2_O) or dopamine (10^-6^M) for 1 hour, then fixed and stained for cell nuclei (DAPI), flotillin-1, CD71, and either ECL2 CCR5 or NT CCR5. Representative images of each stain are found in **Figures 5A, B**. Flotillin-1 and CD71 are expressed in lipid raft (114) or non-raft areas (115), respectively, and were used to differentiate lipid raft regions from non-raft regions. Changes in the colocalization of each CCR5 conformation with Flotillin-1 and CD71 were defined using Pearson’s correlation coefficient (PCC). Using PCC, correlation values above 0.3 indicate varying degrees of colocalization, while those below 0.3 indicate no colocalization. Colocalization between Flotillin-1 and CD71 was used as a positive control for accurate staining, as lipid raft and non-lipid raft should be detected as distinct regions within the macrophage membrane. To ensure the accuracy of these analyses, control studies were performed to show that dopamine treatment did not alter the expression of either CD71 or Flotillin-1, or the colocalization of these markers (**Supplementary Figures 4A, B**). In addition, PCC between Flotillin-1 and CD71 did not change with dopamine treatment, indicating no dopamine-mediated change in colocalization between these markers (**Supplementary Figure 4C**). Image analysis of CCR5 and lipid raft of non-raft PCC showed colocalization of both ECL2 CCR5 and NT CCR5 with Flotillin-1, indicating that both conformations of CCR5 are found in lipid rafts. Dopamine did not change the PCC of either NT CCR5 or ECL2 CCR5 relative to vehicle, and there was no difference between NT CCR5/Flotillin-1 colocalization and ECL2 CCR5/Flotillin-1 colocalization in either condition (**Figure 5C**). In contrast, PCC did not show colocalization between NT CCR5 and CD71, although this was shown for ECL2 CCR5. Dopamine also had no effect on colocalization (or not) with CD71 (**Figure 5C**). This corroborates prior studies showing distinct CCR5 conformations differentially concentrate in lipid rafts (75). Overall, these data indicate that distinct conformations of CCR5 segregate differently within the plasma membrane, but that dopamine does not appear to have any impact on this process at the time point examined.

### Dopamine Increases HIV Replication and NT CCR5 in Human Microglia

**Figure 6:**
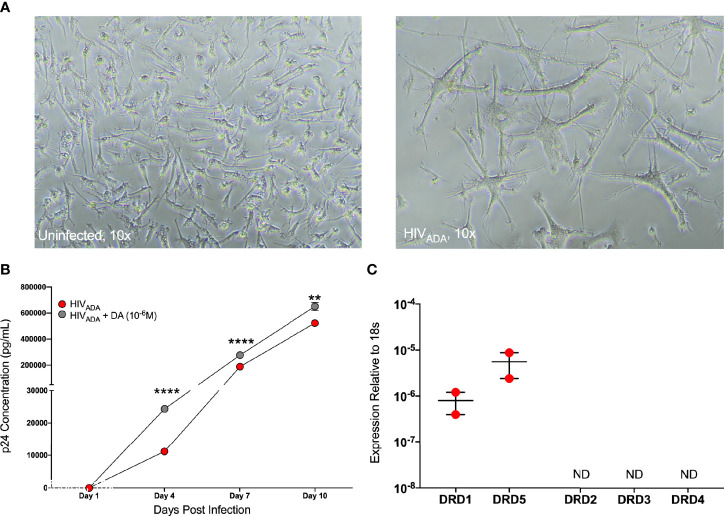
iMicroglia are infectable with HIV and dopamine increases HIV replication. iMicroglia were inoculated with 1 ng/mL of HIV_ADA_ or vehicle and concurrently treated with vehicle (diH_2_O) or dopamine (10^-6^M). **(A)** Representative brightfield images show uninfected or HIV-infected iMicroglia at 7 days post infection, showing high levels of cell fusion and giant cell formation in infected cultures relative to healthy ramified microglia in mock-infected cultures. **(B)** Analysis of supernatant p24 levels over 10 days post-infection in one iMicroglia line (WT6) shows increasing viral replication over time. The p24 levels in dopamine-treated cultures were significantly higher than those in HIV-infected cultures treated with vehicle at every time point examined, indicating that dopamine does increase HIV infection in microglia (multiple t-tests corrected for multiple comparisons using the Holm-Sidak method, Day 4, ****p < 0.0001, t=53.35, df=4, HIV SEM=241.73, HIV+DA SEM=51.5; Day 7, ****p < 0.0001, t=27.52, df=4, HIV SEM=287.37, HIV+DA SEM=3189.41; Day 10, **p = 0.002, t=7.07, df=4, HIV SEM=2294.24, HIV+DA SEM=17989.88). **(C)** Quantitative RT-PCR analysis of dopamine receptor expression in this line of iMicroglia (WT6) showed that these cells express the D1-like dopamine receptors, DRD1 and DRD5, but not D2-like receptors.

The concentrations of dopamine induced by substance abuse are greatest in the CNS, where the major myeloid populations include microglia as well as several distinct types of macrophages (116, 117). Microglia express dopamine receptors and can respond to dopamine (118–120), therefore we examined whether dopamine affects HIV infection in microglia similarly to macrophages. To do this, we used iPSC-derived microglia (iMicroglia) with very similar gene expression to primary human microglia (86). iMicroglia were inoculated in triplicate with vehicle or HIV_ADA_ (1 ng/mL) treated concurrently with vehicle (diH_2_O) or dopamine (10^-6^M) for 24 hours, then washed and cultured until 10 days post-inoculation. Representative brightfield images show uninfected or HIV-infected iMicroglia at 7 days post infection, showing high levels of cell fusion in infected cultures relative to healthy ramified microglia in mock-infected cultures (**Figure 6A**). Analysis of supernatant p24 levels in iMicroglia indicates increasing viral replication over time. Levels of p24 in dopamine-treated cultures were significantly higher than those in vehicle-infected cultures at every time point examined, indicating that dopamine does increase HIV infection in microglia (**Figure 6B**). Analysis of dopamine receptor expression in this line of iMicroglia showed that these cells express the D1-like dopamine receptors, DRD1 and DRD5, but not D2-like receptors (**Figure 6C**).

**Figure 7:**
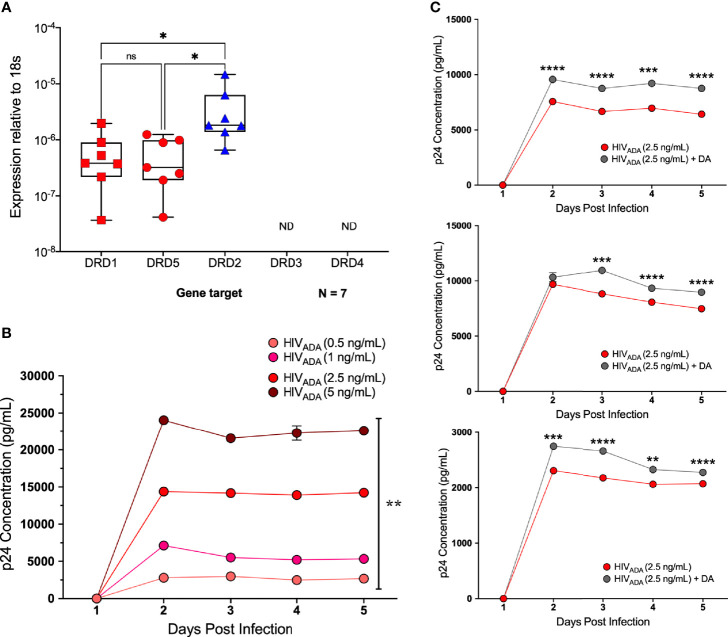
C06 human microglia are infectable with HIV and dopamine increases HIV replication. **(A)** Quantitative RT-PCR detected mRNA for DRD1, DRD2, and DRD5 in C06 cells (N=7), and DRD2 expression is higher compared to DRD1 and DRD5 (Kruskal-Wallis test, n = 7, **p = 0.0048, Dunn’s multiple comparisons test, DRD1 *vs.* DRD2, *p = 0.0331, DRD5 *vs* DRD2, *p=0.0175). **(B)** C06 cells were infected with a range of concentrations of HIV_ADA_ (0.5, 1, 2.5, and 5 ng/ml) for 5 days, and analysis of p24 levels shows significant changes in response to different infection levels at each day post-infection (rmANOVA, Day 2, ****p<0.0001, F (1.208, 2.417) = 2992, HIV 0.5 SEM=16.05, HIV 1 SEM=49.75, HIV 2.5 SEM=199.2 HIV 5 SEM=223.17; Day 3, ****p<0.0001, F (1.270, 2.539) = 2429, HIV 0.5 SEM=23.36, HIV 1 SEM=125.7, HIV 2.5 SEM= 241.63, HIV 5 SEM=171.17; Day 4, **p= 0.0031, F (1.024, 2.047) = 292, HIV 0.5 SEM=122.89, HIV 1 SEM=60.18, HIV 2.5 SEM=147.54, HIV 5 SEM=983.1; Day 5, ***p= 0.0004, F (1.052, 2.104) = 1825, HIV 0.5 SEM=42.66, HIV 1 SEM=52.72, HIV 2.5 SEM=31.0, HIV 5 SEM=417.7). **(C)** Representative p24 analysis in 3 C06 passages demonstrating increased HIV replication in dopamine-treated (10^-6^ M), HIV infected (HIV_ADA_ 2.5 ng/ml) cells compared to cells only infected with HIV (multiple t-tests corrected for multiple comparisons using the Holm-Sidak method, top infection: Day 2, ****p < 0.0001, t=13.47, df=4, HIV SEM=132.0, HIV + DA SEM=67.31; Day 3, ****p < 0.0001, t=26.19, df=4, HIV SEM=47.06, HIV + DA SEM=63.88; Day 4, ***p = 0.0018, t=7.355, df=4, HIV SEM=294.12, HIV + DA SEM=84.26; Day 5, ****p < 0.0001, t=18.51, df=4, HIV SEM=114.5, HIV + DA SEM=53.27; middle infection: Day 2, p > 0.05, HIV SEM=63.46, HIV + DA SEM=414.56; Day 3, ***p =0.0002, t=12.96, df=4, HIV SEM=89.97, HIV + DA SEM=137.34; Day 4, ****p < 0.0001, t=18.84, df=4, HIV SEM=17.89, HIV + DA SEM=64.83; Day 5, ****p < 0.0001, t=28.65, df=4, HIV SEM=37.37, HIV + DA SEM=36.39; bottom infection: Day 2, ***p=0.0002, t=12.95, df=4, HIV SEM=27.99, HIV + DA SEM=19.09; Day 3, ****p < 0.0001, t=26.98, df=4, HIV SEM=3.3, HIV + DA SEM=17.64; Day 4, **p = 0.009, t=4.743, df=4, HIV SEM=26.47, HIV + DA SEM=49.53; Day 5, ****p < 0.0001, t=18.35, df=4, HIV SEM=4.94, HIV + DA SEM=9.99). ns, not significant.

To examine whether dopamine-mediated changes in CCR5 in microglia are similar to macrophages, we chose to use a more tractable system, the C06 microglial cell line. These cells exhibit microglia-like morphology and express key microglial surface markers including CD11b, TGFβR, and P2RY12 (87). Gene expression analysis demonstrates that these cells also express dopamine receptors; DRD1, DRD5, and DRD2, and there is significantly higher expression of DRD2 compared to DRD1 and DRD5 (**Figure 7A**). Unlike hMDM and iMicroglia, C06 cells actively replicate, so they were infected with a range of concentrations of HIV_ADA_ (0.5, 1, 2.5, and 5 ng/ml) to define the optimal conditions for HIV infection in these cells. Cultures show an initial burst of replication followed by a steady level of replication over time, with significantly different levels of p24 production in response to different infection levels (**Figure 7B**). Differences in infection dynamics relative to hMDM and iMicroglia are likely due to the fact that these cells divide. Infection with 2.5 ng/ml HIV_ADA_ showed the widest assay window, so the C06 cells were inoculated with this concentration of HIV and concurrently treated with vehicle (diH_2_O) or dopamine (10^-6^M). Analysis of p24 concentrations in infections of 5 distinct passages of C06 cells shows that dopamine increased the amount of HIV infection in C06 microglia at two to five days post-infection compared to vehicle treatment, with 3 representative infections shown in **Figure 7C**. Analysis of different concentrations of dopamine (10^-6^M - 10^-9^M), show that only dopamine at 10^-6^M increases p24 levels (**Supplementary Figure 5**), unlike studies in hMDM showing an effect of dopamine at 10^-8^M and above (55).

**Figure 8:**
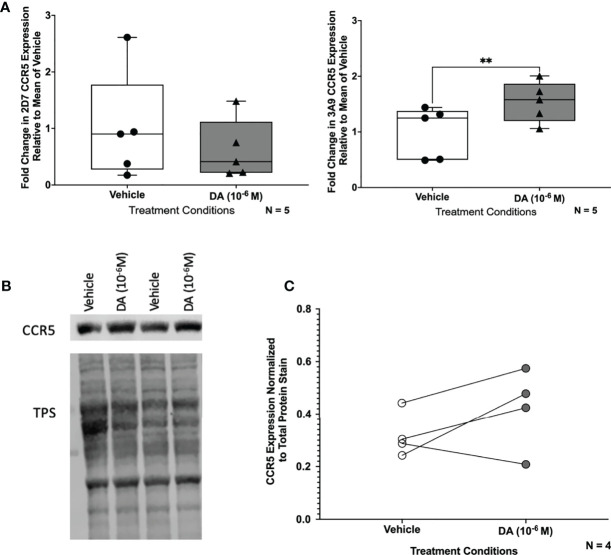
Dopamine increases NT CCR5 in C06 human microglia. **(A)** Change in CCR5 expression relative to the mean of vehicle-treated (diH_2_O) C06 cells demonstrates that dopamine increases NT but not ECL2 CCR5, similar to what we found in hMDM (N=5) (ECL2 CCR5, Paired t-test, n=5, Dopamine, p=0.1818, t=1.614, df=4; NT CCR5, Paired t-test, n=5, Dopamine, **p=0.0043, t=5.837, df=4). C06 cells were also treated with dopamine (10^-6^ M) for 1 hour and probed for pan NT CCR5. A representative blot is shown in **(B). (C)** Pooled data showing fold change in C06 cells from 4 passages relative to the vehicle condition is shown with CCR5 normalized to total protein stain. Although this did not reach significance (Paired t-test, n=4, Dopamine, p=0.2182, t=1.553, df=3), three out of four passages show an increase in NT CCR5 with dopamine relative to vehicle.

After demonstrating that dopamine increased HIV infection in C06 cells, we examined whether dopamine could alter CCR5 conformations, similar to hMDM. Pooled data from flow cytometric analyses of 5 passages of dopamine-treated microglia showed that dopamine significantly increased the percentage of surface NT CCR5 but not ECL2 CCR5 at 1 hour (**Figure 8A**). Similar data were seen by Western blot analysis using an antibody that targets the entire N-terminal region (**Figure 8B**, full blot in **Supplementary Figure 3**). Analysis of the pooled Western data show that dopamine increased NT CCR5 in 3 out of 4 passages, but this did not reach significance (**Figure 8C**). These data indicate that dopamine also increased HIV infection in microglia and that these effects may also be mediated by dopamine-induced changes in the surface conformation of CCR5.

## Discussion

Substance abuse is a major comorbidity in HIV infection, and rates of HIV infection among substance abusers are up to twenty-two times higher than in the general public (8–15). Greater disparities are seen among older adults, a significant portion of the infected population (121, 122). Substance abuse is a significant issue during HIV infection, as it substantively worsens clinical outcomes and accelerates systemic disease even in infected individuals on suppressive ART (17, 83, 123–132). This is also true in the CNS, where substance abuse can still promote neuroinflammation, altering the progression of neuropathology and increasing the risk of neuropsychiatric comorbidities and cognitive decline (16–26). Substance abuse likely mediates these effects by dysregulating immune function and increasing HIV replication in CNS myeloid cells such as macrophages and microglia (17, 18, 29–31, 133), which are primary drivers of HIV neuropathogenesis (32–35). Previous data from our lab shows that dopamine, which is increased by the use of all addictive substances, enhances both HIV infection and inflammatory cytokine production in primary human macrophages (50, 54–56, 60).

Infection of myeloid cells requires the chemokine receptor CCR5, and this receptor is also required for dopamine-mediated increases in HIV entry into these cells (55, 83). The interaction of HIV with CCR5 is mediated by the envelope protein, gp120, which generally binds to sites in the N-terminal (NT) and second extracellular loop (ECL2) regions of the receptor. Both gp120 and endogenous CCR5 ligands have different affinities for each region, meaning that binding affinity varies depending on the region(s) exposed and available for binding (134, 135). Binding site availability depends on receptor conformation, which is heterogeneous across the plasma membrane. This is why some antibodies to CCR5 more effectively inhibit chemokine binding and function (136), while others more successfully inhibit HIV infection (75). The associations between drug-related behaviors, dopamine and CCR5 (70) suggest dopamine-mediated shifts in the expression of distinct CCR5 conformations could alter the functions of this receptor. Thus, dopamine levels induced by substance abuse could, at least in part, promote the development of neuroHIV by increasing HIV entry and also by interfering with CCR5-targeted antiretroviral therapies such as the CCR5 inhibitor, maraviroc.

Maraviroc acts by blocking the interaction between CCR5 and gp120 through allosteric inhibition, stabilizing a CCR5 conformation inducing inefficient ligand binding at the ECL2 binding site (137). This allosteric inhibition is less effective at blocking binding activity that primarily targets the N-terminal region (138), resulting in diminished effectiveness of maraviroc against strains of HIV that have stronger interactions with the CCR5 N-terminus (139–141). This suggests that dopamine induced shifts in the expression of NT CCR5 would reduce maraviroc efficacy. Our data support this hypothesis, confirming that dopamine increases HIV infection in myeloid cells and alters the effectiveness of this inhibitor. Dopamine had multi-modal effects on maraviroc in hMDM from 12 donors, enhancing (5/12 donors), inhibiting (5/12 donors), or having no effect (2/12 donors) on maraviroc efficacy in these studies. When comparing the effect of dopamine on maraviroc efficacy to the effect of dopamine on infection, the hMDM in which dopamine reduced the efficacy of maraviroc showed an average of a 4.67-fold increase in p24 concentrations in response to dopamine alone. In contrast, the hMDM in which dopamine enhanced the efficacy of maraviroc only showed an average of a 1.48-fold increase in response to dopamine. The hMDM from donors in which dopamine did not increase p24 levels also showed that dopamine enhanced maraviroc efficacy. Thus, hMDM in which dopamine inhibited the effectiveness of maraviroc also had a much greater dopamine-mediated increase in HIV infection, suggesting that the mechanism by which dopamine increases HIV entry is connected to the impact of dopamine on maraviroc. This also suggests that dopamine responsiveness varies between donors, and that individuals with the greatest responsiveness to dopamine would also see the most detrimental effects of dopamine on maraviroc- mediated inhibition of HIV infection.

HIV entry and replication increase with CCR5 density (142), and CCR5 is necessary for dopamine to increase HIV entry (55), suggesting that dopamine-mediated changes in CCR5 are at least a part of the mechanism by which dopamine increases HIV infection. R5-tropic strains of HIV originating in the brain have increased affinity for CCR5 (143, 144), potentially due to increases in CCR5 binding efficiency mediated by additional atomic contacts at the gp120-NT CCR5 interface (145, 146). Thus, across a population, an increase in the number of hMDM expressing CCR5 would enhance viral spread. This hypothesis correlates well with our previous studies showing that dopamine increases the amount of HIV entry and replication by increasing the number of HIV-infected cells (54, 55). The data in this study further support this hypothesis, showing that populations of hMDM and microglia exposed to drug-induced dopamine levels have an increased number of cells expressing greater levels of CCR5 on the cell surface. An initial increase is observed in NT CCR5 and not ECL2 CCR5, corroborating previous findings (55, 83). In addition, dopamine treatment significantly increased expression of both conformations of CCR5 at 48 hours, with a greater increase in expression than observed at the 1 hour timepoint. This indicates that dopamine has a greater effect on multiple CCR5 populations over a longer period of time, potentially explaining the dopamine-mediated changes in HIV replication seen at 3 days.

It is important to note that changes in both NT and ECL2 CCR5 were observed at 1 hour using immunofluorescence assays and high throughput imaging, suggesting that the flow cytometry assays used were not sensitive enough to detect changes in ECL2 CCR5 at 1 hour. This could be due to the effects of dopamine affecting ECL2 CCR5 levels on a smaller number of macrophages. An additional consideration highlighted by the flow cytometry assays is that the effects of dopamine on CCR5 were not uniform across the population, increasing expression of NT CCR5 from 3 to 90% in hMDM derived from different individuals. On average, the significant increases in CCR5 were relatively modest, approximately 9% (NT CCR5) at 1 hour, and approximately 12% (ECL2 CCR5) and 16% (NT CCR5) at 48 hours. However, even small increases in surface CCR5 have been shown to have a robust functional impact. Both *in vitro* and *in vivo* studies show progression of HIV infection is heavily dependent on CCR5 expression, with increases in both CCR5 expression and the percentage of CCR5-expressing cells correlating with immune cell activation, plasma viremia, and disease progression (81, 82, 147). In HIV infection of human macrophages and microglia *in vitro*, 50 – 60% increases in surface CCR5 increased HIV entry by 588 – 985% (79), while increasing CCR5 expression approximately 300%, from 7 x 10^2^ to 2 x 10^3^ CCR5 molecules in a HeLa indicator cell line increased HIV infectivity titers more than three orders of magnitude (80). Similarly, decreasing surface CCR5 by approximately 20% reduces viral fusion and p24 production by 50 – 80% in primary human macrophages (148). These data and our previous studies have shown that the effect of dopamine varies widely between donors, but that on average, dopamine increases HIV entry and replication by between 100 – 200% (54–56) which is in line with the smaller increases in CCR5 observed in response to dopamine in these studies. Overall, this suggests that a) dopamine-mediated changes in CCR5 could be the mechanism by which dopamine increases HIV infection and b) if the variation in the impact of dopamine on myeloid susceptibility to HIV infection is connected to the dopaminergic impact on CCR5, it is likely to vary between individuals, similarly to the dopamine-mediated influence on maraviroc efficacy.

Our correlations suggest the dopamine-mediated changes in CCR5 are associated with expression of multiple types of dopamine receptors. The data show a positive correlation between D1-like receptors and CCR5 expression, and increased expression of CCR5 transcripts in hMDM with detectable DRD1 expression. There is also a negative correlation between CCR5 and expression of DRD3, and hMDM with no DRD3 or DRD4 show high levels of CCR5. This suggests that both the activity of D1-like receptors and the lack of activity of DRD3/DRD4 influence CCR5 expression and conformational rearrangements. This is supported by the larger increase in NT CCR5 expression in the C06 microglial cell line, which expresses DRD1 but no DRD3 or DRD4. This is also supported by studies indicating that D1-like dopamine receptors are the most prevalent subtype on macrophages, and that these receptors are likely the primary mediators of dopamine signaling in this cell type (56). In contrast to this hypothesis, others have shown that D1-like agonists reduce CCR5 expression in THP-1 cells, but these differences may be due to the distinct expression levels of dopamine receptors in this cell type, as they express high levels of DRD4 while hMDM and other myeloid cells do not (83). While many types of immune cells express dopamine receptors (52), the nature and the relative proportions of distinct CCR5 populations may vary in other cell types, meaning that the dopamine-mediated effects on HIV infection could be unique to myeloid cells (75, 149).

The specific mechanisms by which dopamine could induce changes in CCR5 conformation are not clear, but distinct CCR5 conformations preferentially localize to lipid rafts and are dependent on cholesterol in order to facilitate productive HIV infection (75, 150). Further, substances of abuse can increase the localization of GPCRs to lipid rafts (110, 151), suggesting dopamine might induce changes in the localization of specific CCR5 conformations. However, the data show that while NT CCR5 is preferentially localized to lipid rafts, this effect is not dependent on dopamine levels. Dopamine could also alter CCR5 conformation by mediating post-translational modifications, such as glycosylation, phosphorylation, or palmitoylation, as these have been shown to alter the HIV entry process and influence CCR5 binding to both chemokines (152–154). Dopamine also regulates the internalization and recycling of G-protein coupled receptors other than dopamine receptors (155), and different CCR5 conformations exhibit distinct sensitivities to endocytosis inhibition (74). Thus, dopamine could potentially promote changes in CCR5 internalization that alter the expression of CCR5 populations on the cell surface. Similarly, dopamine-mediated changes in CCR5 conformations could alter the proportion of cells coupling to different signaling pathways activated by this receptor, as there is select sensitivity of CCR5 conformations to different G proteins (156, 157). Our previous data show that D1-like dopamine receptors in macrophages act primarily through calcium release mediated by G_q/11_ (56), which is also a major signaling mechanism for CCR5. Thus, crosstalk between D1-like receptors and CCR5 signaling is another possible mechanism for interaction between these receptor systems. This is important as CCR5 inhibitors, such as TAK-779 and maraviroc, have different affinities for CCR5 that depend on G protein coupling. Thus, dopamine-mediated changes in CCR5 that lead to differential G protein association could affect the potency and efficiency of these inhibitors in blocking gp120 binding (158).

There are a number of limitations that should be considered in regard to these data, many of which are associated with the inherent variability among primary immune cells from different donors (106, 107, 159). While this variability is expected, it often interferes with standard statistical analysis and necessitates larger n to properly evaluate results. The variance is likely due to genetic factors, as differences in infection levels (93, 160) and the response to dopamine are very high between donors, and in these studies maraviroc showed variable effectiveness across donors. It is possible that these differences could create experimental artifacts in some donors due to a smaller assay window. For example, in the flow cytometry data, donors with high baseline CCR5 showed less impact of dopamine or the positive control (IL-10) on CCR5 expression. Similarly, the effects of dopamine on maraviroc may have been more observable in donors with higher baseline infection due to the larger potential range of changes to infection, an artifact of the culture system in which there are only a limited number of cells to infect. Another caveat is that the hMDM in these studies only have a small amount of epidemiologic data associated with them, precluding analysis of a number of factors that differ between donors that may influence HIV infection or hMDM function. In particular, ongoing substance abuse or dopaminergic medications may influence dopamine levels in the periphery (161–163), potentially influencing expression or sensitivity of hMDM dopamine receptors. Thus, some of the inter-donor variability, as well as the lack of correlation in some analyses, may be attributed to changes in dopamine-responsiveness due to exogenous drugs or therapeutics.

Despite these caveats, we have previously published a consistent effect of dopamine on HIV infection of primary human macrophages derived from these sources (54–56). Further, the dopamine-mediated increases in HIV infection were also seen in the iPSC-derived microglia and C06 microglial cell line, and the C06 cells also showed the dopamine-mediated changes to CCR5. Taken with the need for a relatively large n, this suggests that while the effects we are observing are consistent, the magnitude is modest and can therefore be obscured due to donor variability and the detection limits of the assays. A final caveat regarding the use of primary cells is that many of the studies occurred sequentially using human blood that is de-identified and of limited supply, so it was not possible to perform all of the experiments in each donor. Future analyses based on these studies will be designed to better accommodate running all assays for a particular study in cells from each donor. Another technical caveat to consider is the capacity for CCR5 antibodies to detect different CCR5 conformations. Our data show a differential average surface expression of ~40% for the ECL2 CCR5 (2D7) *vs.* an ~80% average expression for the NT CCR5 (3A9). While these studies were performed using well-established antibodies validated for this type of assay, using only one CCR5 antibody might underestimate the total CCR5 cell surface expression level under certain conditions. Future studies investigating CCR5 should use multiple antibodies – although this will necessitate the generation of a large number of more effective antibodies – and should consider the existence of multiple conformations during analysis (74).

Overall, these data indicate that induction of an increased concentration of extracellular dopamine may be a common mechanism by which different classes of abused substances could drive neuroHIV. Dopamine-mediated increases in HIV entry may be driven by changes in the diversity of CCR5 populations on the surface of myeloid cells. In addition to increasing the general susceptibility to HIV infection, these changes may alter the effectiveness of the CCR5 inhibitor maraviroc. This demonstrates a critical need to better define the specific neurobiology driving neuroHIV in infected substance abusers, and to specifically evaluate the efficacy of ART drugs in this unique environment. To accommodate this, studies should consider targeting specific conformations of CCR5, or developing bivalent ligands, such as dual DR/CCR5 antagonists, that could block possible signaling pathways that promote HIV infectivity. These data also suggest novel therapeutic approaches for a variety of other pathologies, such as multiple sclerosis, atherosclerosis and several types of cancers, that may be impacted by dopamine-driven CCR5 expression. Future studies in this area will be facilitated through further examination of human primary macrophages with more detailed epidemiologic data, as well as through the use of iPSC-derived myeloid cells. These studies show for the first time that dopamine increases HIV infection in iMicroglia, and future studies using iMicroglia and iMacrophages will be extremely valuable as a more tractable platform in which to perform more complex molecular assays. Use of both macrophages and microglia is important because myeloid populations in the CNS are transcriptionally related (164), but microglia and macrophages are distinct cell types (165), and infection of both populations is central to the development of neuropathology (32–35, 166–170).

More broadly, these data further emphasize the role of dopamine as an immunomodulatory factor in a variety of pathological and homeostatic conditions. Many dopaminergic drugs are currently in use as treatment for a variety of disorders, and concentrations of dopamine induced by both substance abuse and these therapeutics have both subtle and robust effects on a wide array of immune functions. Thus, future therapeutic strategies based on development and repurposing of these drugs in order to manipulate dopaminergic immunology would likely be beneficial for not only neuroHIV but many diseases in which CCR5 plays a role. Returning to neuroHIV, these data highlight the critical need for studies that define more precisely the relationship between substance abuse and progression of neuroHIV. Further studies in this area are essential to the development of specific strategies, ART combinations and other targeted therapeutics that are efficient and effective at blocking the development of neuropathology specifically in the vulnerable population of HIV-infected substance abusers.

## Data Availability Statement

The original contributions presented in the study are included in the article/**Supplementary Material**. Further inquiries can be directed to the corresponding author.

## Ethics Statement

Ethical review and approval was not required for the study on human participants due to the deidentified nature of the material, but all human material was obtained in accordance with the local legislation and institutional requirements set out by the Institutional Review Board of Drexel University. Written informed consent for participation was not required for this study in accordance with the national legislation and the institutional requirements.

## Author Contributions

SM, YR, and PG contributed to the design and conception of the study. SM, EN-B, YR, KR, HJ, MO’C, and PG designed and analyzed the experiments, and SM, EN-B, YR, KR, HJ, and MO’C performed the experiments. EH and PG helped supervise the project. SM and PG performed the statistical analyses and wrote the manuscript. PG was responsible for the final approval of the submitted version. All authors contributed to the article and approved the submitted version.

## Funding

This work was supported by grants from the National Institutes of Drug Abuse, DA039005 and DA049227 (PJG), the W.W. Smith Charitable Trust Foundation Grant A2003 (PJG), the Brody Family Medical Trust Fund (SM), the Clarkston Scholarship program (HJ) and the Drug, Discovery and Development program and the Department of Pharmacology and Physiology at Drexel University College of Medicine. NIH 1RO1AI106482-01 to EKH, and EAN is a trainee of the T32 grant “Interdisciplinary and Translational Research Training in neuroAIDS” from the National Institute of Mental Health (MH079785).

## Conflict of Interest

The authors declare that the research was conducted in the absence of any commercial or financial relationships that could be construed as a potential conflict of interest.
