# Supplementary material for: Dopamine Levels Induced by Substance Abuse Alter Efficacy of Maraviroc and Expression of CCR5 Conformations on Myeloid Cells: Implications for NeuroHIV
Source: Front Immunol. 2021 May 19;12:663061. doi: 10.3389/fimmu.2021.663061 (PMC8170305; doi:10.3389/fimmu.2021.663061)

Supplementary Material

## Supplementary Methods

- 1. **CCR5 Immunofluorescent Analysis**

For CCR5 immunofluorescent analysis, after the images were acquired on the CX7, they were quantitatively analyzed using the HCS Studio software, using the Thermo Scientific Cellomics Colocalization Bioapplication, and specific parameters are included in Supplementary Table 1. These images had donor-specific manual alterations in Image Settings to correct any background. The nuclear and the whole-cell stains were gated by area and average intensity (Object.Area.Ch1, Object.AvgIntensity.Ch1, ObjectCh2.Area.Ch2, and ObjectCh2.AvgIntensity.Ch2). Both CCR5 stains were gated by average intensity (ObjectCh3.AvgIntensity.Ch3 and ObjectCh4.AvgIntensity.Ch4). The first colocalization, ROI A, had a focal channel of Wheat Germ Agglutinin and a target channel of 2D7 CCR5 stain. The second colocalization, ROI B, had a focal channel of Wheat Germ Agglutinin and 3A9 CCR5 stain was the target channel.

After the whole plate had been scanned, the colocalization between WGA and the CCR5 markers were quantified by object count and total intensity. The average object count (Mean_ROI_A_Target_I_ObjectCount and Mean_ROI_B_Target_I_ObjectCount) depicted the average number of CCR5 stained objects. The raw object counts were used to find the total number of cells (via WGA) and all of the cells without stain. The total cell count was subtracted by the number of cells without CCR5 to yield the number of cells with CCR5 in that specific well. Then, the number of cells with CCR5 was divided by the total number of cells within that well to enumerate the percentage of cells with CCR5 stain within each well. The average total intensity (Mean_ROI_A_Target_I_ObjectTotalInten and Mean_ROI_B_Target_I_ObjectTotalInten) quantify the intensity of the CCR5 stain that exists within the whole cell stain, WGA. These averages are the total intensity of CCR5 stain normalized by cell count.

- 1. **Immunofluorescent Analysis of Lipid Raft Colocalization with CCR5**

For immunofluorescent analysis, the Thermo Scientific Cellomics Colocalization Bioapplication was used for assay development, and specific parameters are included in Supplementary Table 2. Background correction in each fluorescence channel was performed, and objects were identified based on intensity threshold and segmentation and validated by setting up border object exclusion as well as the minimum and maximum value of object total area, object total intensity, object average intensity and object variable intensity. A region of interest mask was created for each fluorescent channel. For colocalization analysis, 3 regions of interest were created, one for each pair of targets, to define the colocalization between CD71, Flotillin-1 and CCR5. Pearson’s correlation coefficient (PCC) was used to determine changes of colocalization in each region of interest between each target. This correlation coefficient is a widely used statistic that has been previously characterized for use in fluorescent microscopy and can measure the pixel-by-pixel covariance in the signal levels of two colors within an image [1, 2]. Because it subtracts the mean intensity from each pixel's intensity value, PCC is independent of signal levels and signal offset (background), making it both simple and relatively safe from user bias [3]. PCC values above 0.3 indicate various degrees of colocalization, and a value below 0.3 indicates no colocalization [4]. Increased colocalization of epitope-specific CCR5 with flotillin-1 in dopamine-treated hMDM relative to vehicle hMDM will indicate that dopamine increases the percentage of specific CCR5 conformations in lipid rafts. The relative expression of CCR5 was calculated via mean fluorescence intensity (Mean_ROI_A_Target_1_ObjectAvgInten).

References:

1. Manders, E.M.M., F.J. Verbeek, and J.A. Aten, *Measurement of co-localization of objects in dual-colour confocal images.* Journal of Microscopy, 1993. **169**(3): p. 375-382.

2. Adler, J. and I. Parmryd, *Quantifying colocalization by correlation: the Pearson correlation coefficient is superior to the Mander's overlap coefficient.* Cytometry A, 2010. **77**(8): p. 733-42.

3. Dunn, K.W., M.M. Kamocka, and J.H. McDonald, *A practical guide to evaluating colocalization in biological microscopy.* American Journal of Physiology - Cell Physiology, 2011. **300**(4): p. C723-C742.

4. Mukaka, M.M., *A guide to appropriate use of Correlation coefficient in medical research.* Malawi Medical Journal : The Journal of Medical Association of Malawi, 2012. **24**(3): p. 69-71.

**2. Supplementary Tables**

**Supplementary Table 1.** Parameters for Colocalization using High Content Analysis of Immunocytochemistry

| **Condition** | **Value** |
| --- | --- |
| Ch 1 (DAPI) Thresholding: Isodata | 0 |
| Ch 1 (DAPI) Segmentation: Shape | 5 |
| Object.Area.Ch1 | 0 – 508.33 |
| Object.AvgIntensity.Ch1 | 60.12 – 13956.89 |
| Ch 2 (WGA) Smoothing: Uniform | 10 |
| Ch 2 (WGA) Thresholding: Isodata | -0.937 |
| Ch 2 (WGA) Segmentation: Shape | 10 |
| ObjectCh2.Area.Ch2 | 113.27 – 1x10¹² |
| ObjectCh2.AvgIntensity.Ch2 | 976.03 – 65535 |
| Ch 3 (2D7) Smoothing: Uniform | 3 |
| Ch 3 (2D7) Thresholding: Isodata | -0.947 |
| ObjectCh3.AvgIntensity.Ch3 | 264.33 – 65535 |
| Ch 4 (3A9) Smoothing: Uniform | 1 |
| Ch 4 (3A9) Thresholding: Isodata | 0 |
| ObjectCh4.AvgIntensity.Ch4 | 2268.72 – 65535 |
| ROI A Channel | 2 (WGA) |
| ROI A Target I | 3 (2D7) |
| ROI B Channel | 2 (WGA) |
| ROI B Target I | 4 (3A9) |

**Supplementary Table 2.** Parameters for Colocalization using High Content Analysis of Immunocytochemistry

| **Condition** | **Value** |
| --- | --- |
| Ch 1 (DAPI) Smoothing: Uniform | 1 |
| Ch 1 (DAPI) Thresholding: Fixed | 700 |
| Object.Area.Ch1 | 56.6 – 4000.53 |
| Object.AvgIntensity.Ch1 | 1044.08 – 7500.46 |
| Object.VarIntensity.Ch1 | 0 – 32767 |
| Ch 2 (CD71) Smoothing: Uniform | 2 |
| Ch 2 (CD71) Thresholding: Fixed | 200 |
| Ch 2 (CD71) Segmentation: Intensity | 1500 |
| ObjectCh2.Area.Ch2 | 821.52 – 2000.65 |
| ObjectCh2.AvgIntensity.Ch2 | 329.32 – 32767 |
| ObjectCh2.VarIntensity.Ch2 | 0 – 32767 |
| Ch 3 (CCR5) Smoothing: Uniform | 2 |
| Ch 3 (CCR5) Thresholding: Fixed | 200 |
| Ch 3 (CCR5) Segmentation: Intensity | 1500 |
| ObjectCh3.Area.Ch3 | 279.63 – 3000 |
| ObjectCh3.AvgIntensity.Ch3 | 214.20 – 6000 |
| ObjectCh3.VarIntensity.Ch3 | 0 – 32767 |
| Ch 4 (Flotillin-1) Smoothing: Uniform | 2 |
| Ch 4 (Flotillin-1) Thresholding: Fixed | 250 |
| ObjectCh4.Area.Ch4 | 255.36 – 30000 |
| ObjectCh4.AvgIntensity.Ch4 | 410.94 – 1000.99 |
| ObjectCh4.VarIntensity.Ch4 | 22.02 – 32767 |
| ROI A Channel | 4 (Flotillin-1) |
| ROI A Target 1 | 3 (CCR5) |
| ROI A Target 2 | 4 (Flotillin-1) |

1. **Supplementary Figures**

**Supplementary Figure 1. Dopamine Similarly Alters Effectiveness of a Higher Concentration of Maraviroc in HIV-infected hMDM.** Primary human monocyte-derived macrophages (hMDM) from a total of twelve donors were inoculated with HIV_ADA_ (0.5 ng/mL) for 24 hours in the presence of vehicle (diH_2_O or DMSO), dopamine (10^-6^M), maraviroc (MVC) (0.1 μM) or MVC + dopamine (10^-6^M). Infections were maintained in culture for 3 days, at which point supernatants were collected from each well and examined for HIV replication. Responses from each donor are designated with a specific color throughout. **(A)** MVC successfully suppressed viral replication at 1μM, in that MVC significantly decreased p24 levels relative to HIV alone (Paired t-test, n = 12, **p = 0.0074, t=3.277, df=11). **(C)** There was a non-significant but trending bimodal response to dopamine in macrophages treated with 1 μM MVC. Five out of twelve donors (46.7%) showed a dopamine-mediated diminished response to MVC (Paired t-test, n = 5, p = 0.0683, t=2.479), and five out of twelve donors (46.7%) showed an dopamine-mediated enhanced response to MVC (Paired t-test, n = 5, p = 0.0636, t=2.546, df=4), with relatively lower p24 levels in cultures treated with dopamine. Two of the twelve donors (16.6%) showed no response to dopamine in respect to the efficacy of MVC.

**
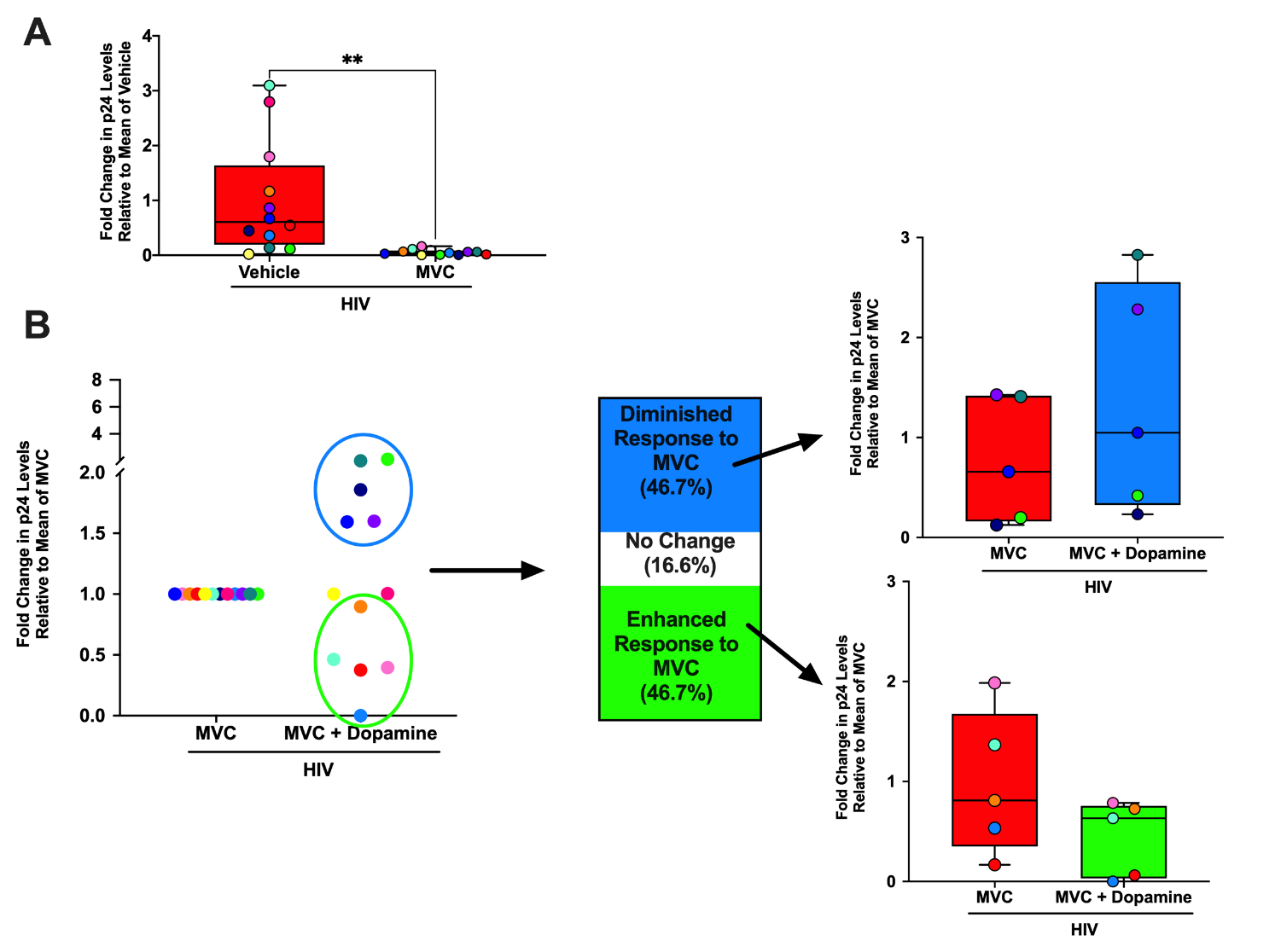
**

**Supplementary Figure 2. CCR5 Correlations with Donor Demographic Data and D2-like Receptors.** Correlational analyses were performed to look at correlations between CCR5 mRNA expression and demographic information. **(A)** There was a trending positive correlation with CCR5 and age (CCR5 vs Age, n = 60, Spearman r, 0.2145, p = 0.0999), **(B)** females had significantly higher CCR5 expression compared to males (Mann-Whitney test, n = 26, * p = 0.0307, sum of (male, female) ranks 571, 807, U=220), and **(C)** CMV+ individuals had significantly higher CCR5 expression compared to CMV- individuals (Mann-Whitney test, n = 19-21, * p = 0.0147, sum of (CMV-, CMV+) ranks 300, 520, U=110). A number of donors lacked expression of one or more dopamine receptors, so the data were reanalyzed for correlations between CCR5 and the **(D)** D2-like (DRD2, 3, 4) dopamine receptors. These analyses showed no correlation between CCR5 and D2-like receptors (CCR5 vs D2-like dopamine receptors, n = 65, Spearman r = 0.2002, p = 0.1099). Further analysis of CCR5 levels in hMDM that did or did not express one of the D2-like receptors showed that **(E)** there was no change in CCR5 expression in groups with or without DRD2 expression (Mann-Whitney test, n = 16 - 40, p = 0.9356, sum of (D2, No D2) ranks 1135, 461, U=315), but hMDM not expressing either **(F)** DRD3 or **(G)** DRD4 had higher levels of CCR5 mRNA than those expressing either dopamine receptor (Mann-Whitney test, n = 17-38, **p = 0.0067, sum of (DRD3, No DRD3) ranks 329, 1211, U=176; Mann-Whitney test, n = 15-39, **p = 0.0086, sum of (DRD4, No DRD4) ranks 278, 1207, U=158).


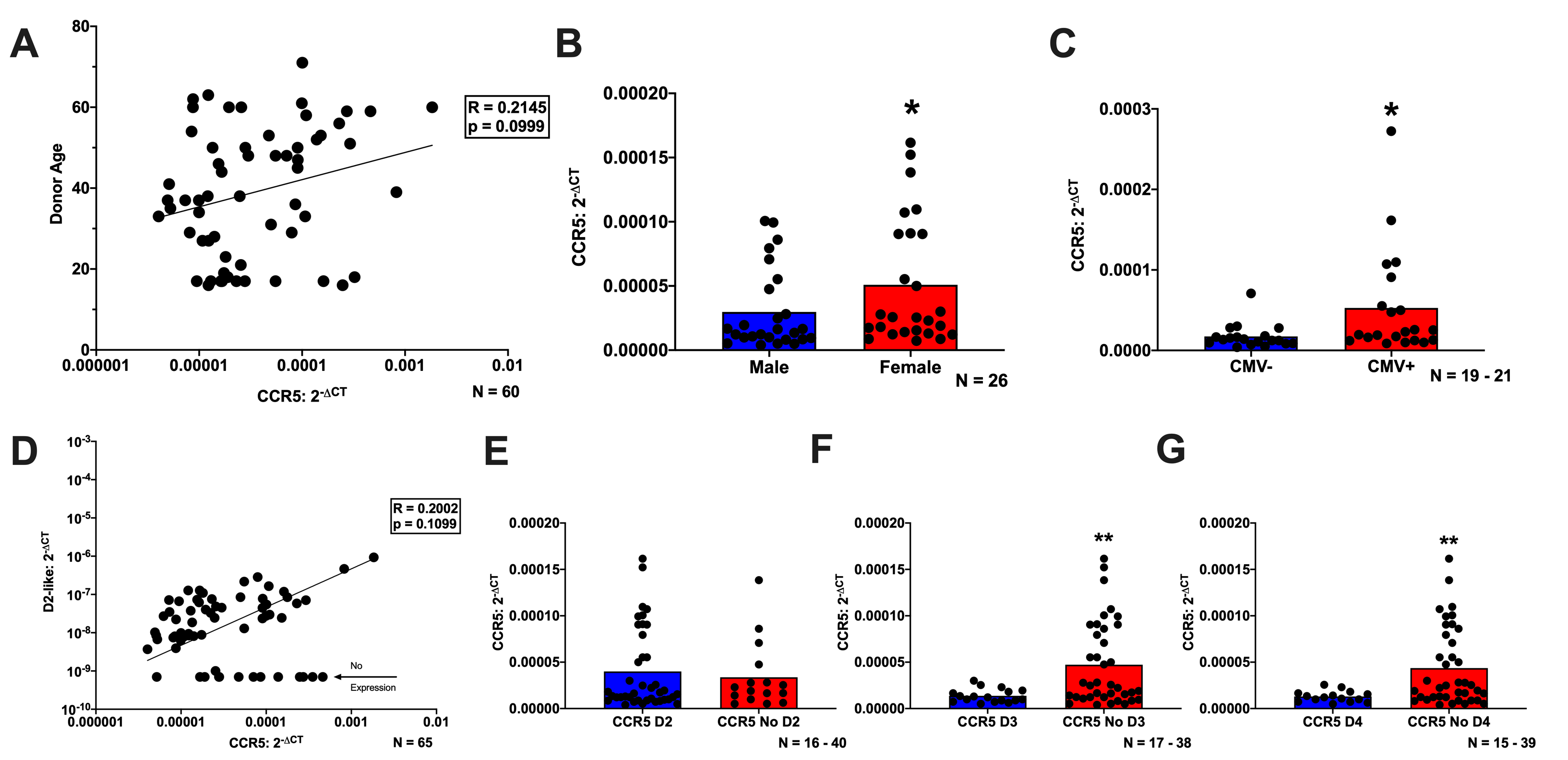


**Supplementary Figure 3. Full Representative CCR5 Western Blots in hMDM and C06 Cells.** Full Western blots of total protein stain (TPS) and CCR5 in **(A)** hMDM and **(B)** C06 microglia. Arrows showing CCR5 band. Molecular weight of CCR5 is 40.6 kDa.


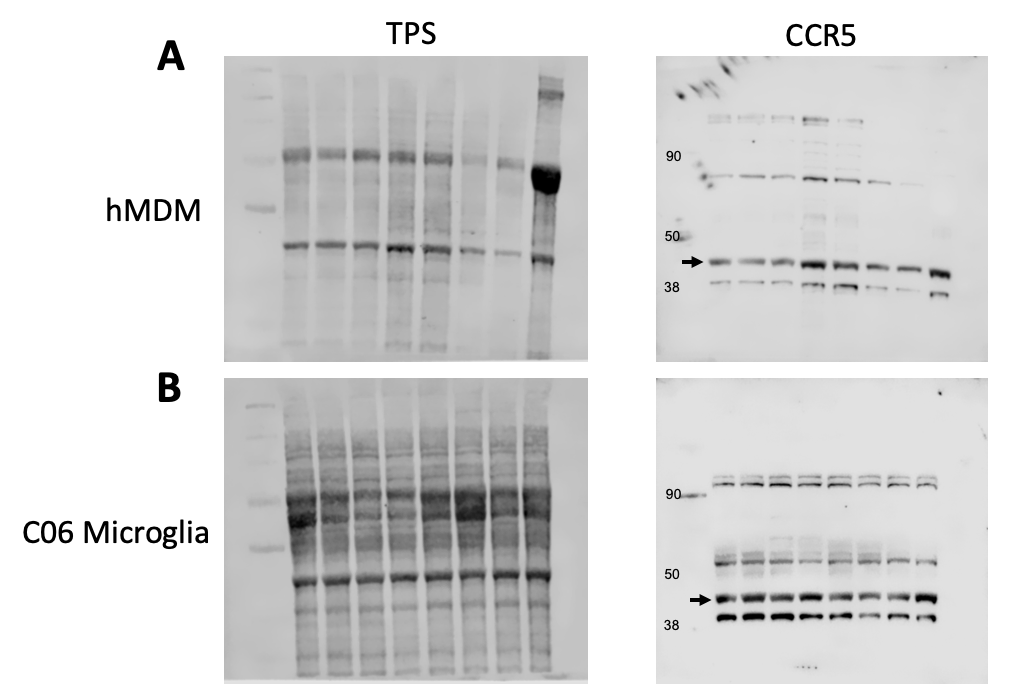


**Supplementary Figure 4. Dopamine Does Not Affect Expression of Lipid Rafts or Non-Lipid Raft Markers or Colocalization of CCR5 to Non-Lipid Raft Regions.** hMDM were imaged at 40X and analyzed using high content imaging with 3 wells per treatment condition. hMDM donors were not the same for each panel analysis. To ensure the accuracy of these analyses, control studies were performed to show that dopamine treatment did not alter the average intensity of either **(A)** flotillin-1 (lipid raft marker) (Wilcoxon test, n = 9, Dopamine, p = 0.7344, sum of (+,-) ranks 19, -26; CD71) or **(B)** CD71 (non-raft marker) (Paired t-test, n = 9, Dopamine, p = 0.0639, t=2.149, df=8). Images were then analyzed to define colocalization between Flotillin-1 and CD71 using Pearson’s correlation coefficient (PCC). Colocalization between Flotillin-1 and CD71 was used as a control for accurate staining, as lipid raft and non-lipid raft should be detected as distinct regions within the macrophage membrane. Using PCC, there was no significant colocalization between Flotillin-1 and CD71 with any treatment, indicating no change in colocalization between these markers (PCC between Flotillin-1 and CD71, vehicle = 0.1611, dopamine = 0.1583).


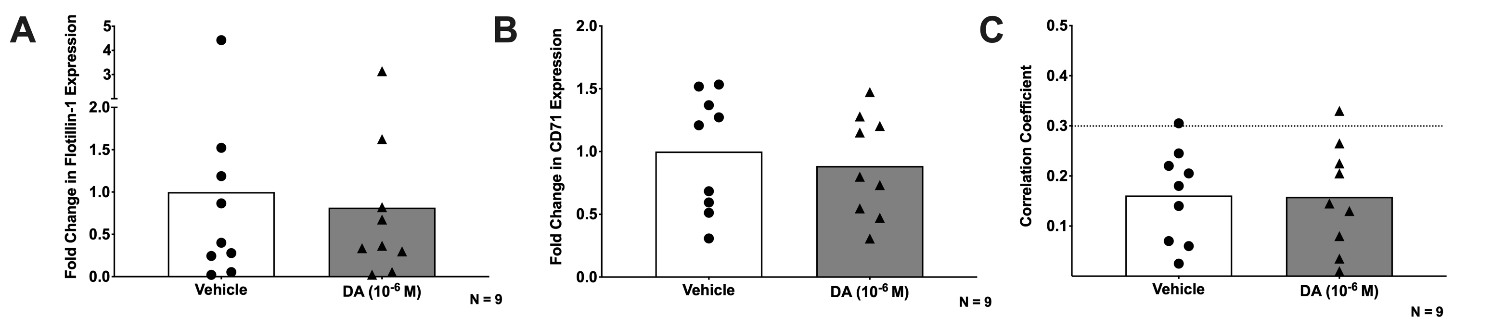


**Supplementary Figure 5. Dose response of dopamine-mediated effects on HIV replication in C06 human microglia.** Representative p24 analysis in one passage of C06 cells demonstrating increased HIV replication in dopamine-treated (10^-6^ M), HIV infected (HIV_ADA_ 2.5 ng/ml) cells compared to cells only infected with HIV, and no significant increases between cells treated with lower concentrations of dopamine (10^-9^ M-10^-7^ M) compared to cells only infected with HIV (multiple t-tests corrected for multiple comparisons using the Holm-Sidak method, 10^-6^M dopamine; Day 2, ** p = 0.0048, t=14.38, df=2, HIV SEM=64.11, HIV + DA^-6^M SEM=46.15; Day 3, ** p = 0.0035, t=16.76, df=2, HIV SEM=107.9, HIV + DA^-6^M SEM=22.46; Day 4, ** p = 0.0037, t=16.3, df=2, HIV SEM=31.54, HIV + DA^-6^M SEM=131.71; Day 5, ** p = 0.0033, t=17.41, df=2, HIV SEM=107.31, HIV + DA^-6^M SEM=62.83).


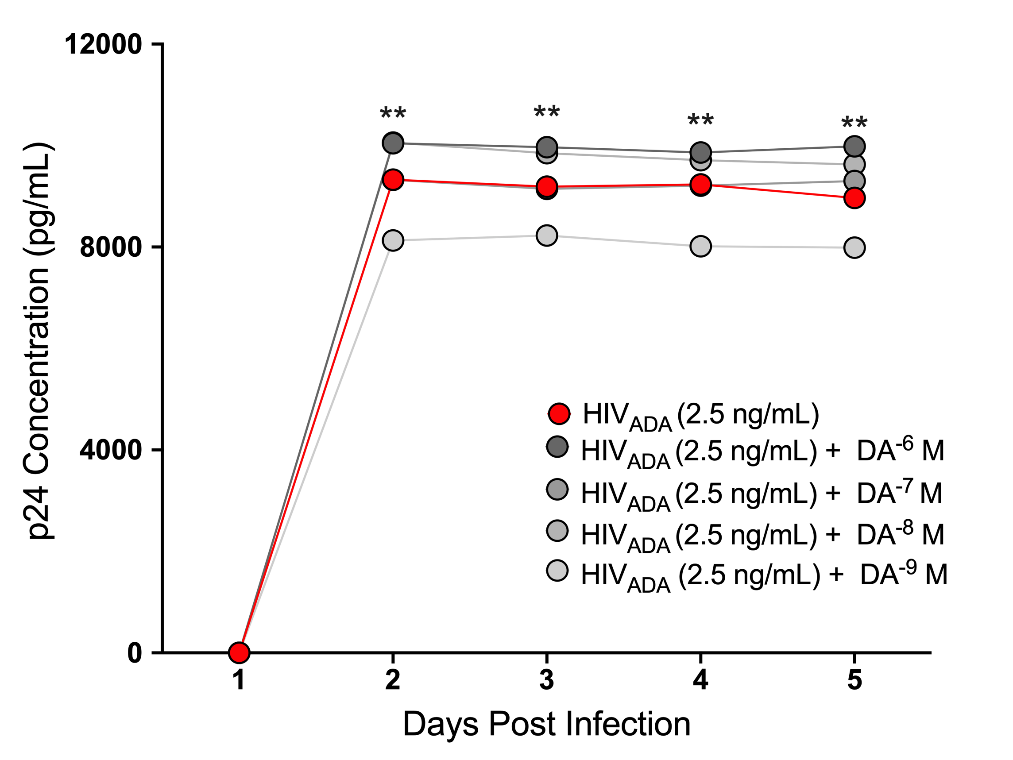

Supplement: Supplementary file 1 [file DataSheet_1.docx]
